# Predicted preference conjoint analysis

**DOI:** 10.1371/journal.pone.0256010

**Published:** 2021-08-26

**Authors:** Sonja Radas, Dražen Prelec

**Affiliations:** 1 Institute of Economics, Zagreb, Croatia; 2 Sloan School of Management, Massachusetts Institute of Technology, Cambridge, Massachusetts, United States of America; Universidad de Murcia, SPAIN

## Abstract

In this paper we propose a new method of eliciting market research information. Instead of asking respondents for their personal choices and preferences, we ask respondents to predict the choices of other respondents to the survey. Such predictions tap respondents’ knowledge of peers, whether based on direct social contacts or on more general cultural information. The effectiveness of this approach has already been demonstrated in the context of political polling. Here we extend it to market research, specifically, to conjoint analysis. An advantage of the new approach is that it can elicit reliable responses in situations where people are not comfortable with disclosing their true preferences, but may be willing to give information about people around them. A theoretical argument demonstrates that predictions should yield utility estimates that are more accurate. These theoretical results are confirmed in four online experiments.

## Introduction

Traditionally, market research begins by asking potential customers for their personal preferences and intentions, and then projects sample data to the entire market. In this paper we propose a different approach: instead of asking respondents about their own likely choices, we ask them to predict the market. This approach has already been successful in political polling, as shown by [1], who demonstrated that voters’ expectations of the election result yield more accurate election forecasts compared to their personal vote intentions. Similarly, a recent test found that election forecasts based on respondents’ judgments of their social circle voting intentions are superior to forecasts based on own intentions [2]. This method of tapping into the “local” wisdom of crowds is complementary to approaches that attempt to neutralize the common-source or shared information bias, which leverage respondents’ predictions of the averaged survey results [3–5].

Here we extend the general approach of tapping peer information to market research, and we use conjoint analysis as our methodological test bed. Conjoint analysis is a method of measuring consumer’s preferences about a product, service, project or policy [6–20]. It is one of the most popular tools in new product development. Conjoint approach assumes that a product can be described as bundles of attributes or features, where each feature can assume a number of pre-specified levels. The total product utility can then be decomposed into feature utilities or ‘part-worths.’ These utilities are estimated from respondents’ choices among offered alternatives.

Although traditional conjoint analysis requests respondents to make personal choices, recently some studies introduced the role of others in choice-making. The authors in [21] achieve better predictive performance by proposing a “group-sourced mechanism”, which mimics real-world context where consumers’ choices are influenced by others around them. Similarly, in [22] it is found that being exposed to peer choices prompts consumers to update their preferences in Bayesian manner. Although these two papers explicitly recognize the influence of peers, preferences are still estimated on the basis of respondents’ personal choices. Our study is different in that we use respondents’ *prediction of peer choices* instead of their personal choices. The closest to our study is [23], in which the authors introduce “securities trading of concepts”, where subjects trade concepts on the basis of their perception of others’ preferences.

Unlike [23] which uses prediction markets methodology, we remain in the domain of conjoint analysis. Instead of asking respondents what they will choose, we ask them to predict the percentage of respondents that will select each option in the set. We refer to these judgments as “peer-predictions.” We use peer-predictions to define peer-choices: the peer-choice in the set of alternatives is the alternative that the respondent thinks will be chosen by the majority of his/her peers. People seem to be quite good at using past observations to predict others’ choices, as shown by [24], who found “evidence that people can learn impressively well from extremely noisy, sparse data to derive reasonable predictions about others’ choices”.

Our hypothesis is that peer-choices yield improved preference share predictions (i.e. market share predictions), relative to predictions based on personal choices. In addition, an important advantage of peer-choice conjoint analysis is that it can elicit reliable responses in situations where people are not comfortable with disclosing their true preferences, but may be willing to give information about people around them. For example, this may happen in research related to health, lifestyle, habits and behavior, etc.

The theoretical argument for our method extrapolates the reasoning of [1] which assumed that peer-predictions result from respondents’ internal (i.e., mental) polling of their social circle. A personal choice may then be interpreted as a degenerate internal poll based on a “circle of one.” A peer prediction is an internal poll based on a larger sample, hence more accurate. We follow this interpretation and adapt it to the binary choice conjoint model, allowing for possible interdependencies among social circle members. It is readily derived that larger social circles will lead to smaller errors in estimation of utilities.

This approach is related to the Bayesian respondent approach in [3]. In that model, respondents are presumed to start with a common prior over the population distribution, and then use their personal preference to compute a posterior by Bayes’ rule. The peer prediction is the expectation of this posterior. Our approach assumes that individuals have sufficiently large and diverse social circles so that their posteriors are insensitive to personal preference (which contributes only one data point to the internal poll).

Our method can be easily combined with traditional conjoint approach in the same survey, by simple addition of peer-prediction questions. In this way, features of traditional conjoint such as segmentation by own preferences can be kept, while adding all the advantages of peer-choice conjoint.

To summarize, in this paper we hope to contribute to the body of research in the following way:

1. We introduce peer-choice in binary choice conjoint analysis instead of personal choice,
2. We empirically test the new approach in four experiments and demonstrate better predictive ability for peer-choice in preference shares.

Four experiments test the hypothesis that peer-choices have better predictive ability. Experiments 1 and 2 use the same type of product (streaming media stick). To make sure results are not dependent on the choice of stimuli, Experiment 3 is performed on a different product (fitness/activity tracker). While Experiments 1, 2, and 3 are within-subjects studies, Experiment 4 is a between-subjects study, where one group of subjects was used for estimation and the other group for validation. All experiments were performed with online panels. In each experiment we compare traditional choice based conjoint (which we refer to as the own-choice model) with the new approach based on peer-choice. We show that statistically the peer-choice conjoint has higher predictive accuracy compared to the traditional conjoint model.

## Materials and methods

In this paper we focus on binary choice based conjoint analysis, where respondents are asked to choose between two presented alternatives. The conceptual framework is the familiar discrete choice model, with respondents choosing the alternative with higher utility.

Within this general setting, we compare two models. The first model is based on the traditional conjoint approach, where utility structure is derived from personal choices that respondents make. We refer to this as the *own-choice model*. In the second model, the utility structure is derived from *peer-choices* that are determined in the following way: (1) each respondent gives peer-predictions for both alternatives in every choice set (by peer-prediction we mean the prediction of what percentage of peers will choose the offered alternatives), (2) if the peer-prediction for alternative A is larger than the peer-prediction for alternative B, we infer that the respondent thinks that A would be chosen by the majority, and therefore we define the peer-choice to be A. In short, the own-choice answers the question “which alternative would you choose?”, while the peer-choice answers the question “which alternative will the majority of your peers choose?”.

### Model 1: Own-choice or traditional conjoint approach

Let *k* = 1,2, denote the two alternatives in the choice set *r*. The utility for an alternative is expressed as a linear combination of features and utilities for each feature (i.e. part-worths). More precisely, for a respondent *i*, the utility can be represented as ${u_{rk}}^{i}\;=\;{w_{rk}}^{i}+{\varepsilon_{rk}}^{i}$, where ${w_{rk}}^{i}$ is the deterministic part of the utility. This deterministic part can be written as ${w_{rk}}^{i}\;=\;\sum_{l}{{\beta_{l}}^{i}}^{\;}{x_{rlk}}^{\;}$, where *β*_*il*_ are utilities for respondent *i* and *x*_*rlk*_ are dummies designating levels of attributes present in the alternative *k*, which is in the given choice set *r*. Let ${z_{r}}^{i}\;$ denote the alternative that respondent *i* chooses in the set *r*. With this convention, the probability of choosing alternative 1 can be written as:

$${P\left({z_{r}}^{i}\;=\;alternative\;1\right)\;=\;P({u_{r1}}^{i}>{u_{r2}}^{i}}_{\;})\;=\;P\left({w_{ri}}^{i}-{w_{r2}}^{i}>{\varepsilon_{r2}}^{i}-{\varepsilon_{ri}}^{i}\right)\;=\;F\left({w_{r1}}^{i}-{w_{r2}}^{i}\right),$$

where errors are assumed to be i.i.d. extreme value distributed. Consequently, *F*, the link function, is the logit.


$$\text{Then},\;P\left({z_{r}}^{i}\;=\;alternative\;1\right)\;=\;\frac{\text{exp}\;(\sum_{l}{{\beta_{l}}^{i}\;}^{\;}{x_{rl1}}^{\;})}{\text{exp}\;\left(\sum_{l}{{\beta_{l}}^{i}\;}^{\;}{x_{rl1}}^{\;}\right)+\text{exp}\;(\sum_{l}{{\beta_{l}}^{i}\;}^{\;}{x_{rl2}}^{\;})}.$$

The Hierarchical Bayesian logit model estimates the vector of individual utilities ${\beta^{i}}_{\;}\;=\;{{(\beta }_{l}}^{i})$ [25–27]. Briefly, the model assumes that the vector of utilities ${{(\beta }_{l}}^{i})$ for any individual *i* is sampled from a multivariate normal distribution, i.e. ${\beta^{i}\;=\;}_{\;}{{(\beta }_{l}}^{i})\;∼N\left(b_{\;},W\right)$ where the vector of means *b* is normally distributed, *b* ~ *N*(*b*_*0*_, *W*_*0*_) with very large variance. In other words, respondents’ preferences are linked by a common multivariate normal distribution. The covariance matrix *W* is assumed to come from an inverse Wishart distribution with a scale matrix that is diagonal with equal diagonal entries. Gibbs sampling or Metropolis-Hastings algorithms are used for drawing posteriors. The resulting values are individual estimates of utilities ${\beta_{l}}^{i}$.

### Model 2: Peer-choice approach

We focus again on respondent *i*. For a choice set *r*, we define the peer-predicted choice to be alternative 1 if the respondent *i* believes that more than 50% of others will choose the alternative 1. Apart from that change, the above model remains the same, except that the meaning of the notation changes. In this model ${z_{r}}^{i}\;$ denotes the peer-choice that is reported by the respondent *i* in the choice set *r*. Notice that the utility ${u_{rk}}^{i}$ is not a personal utility anymore: it is the utility that the respondent *i thinks that others have* for the alternative *k*, where *k* = 1,2. The Hierarchical Bayesian model can be used in the same way as in the own-choice case. The difference is that estimated utilities now reflect the *average utility of others as perceived by the respondent i*, instead of the individual utility of the respondent *i*. We can think about these as expressing the utility structure of the “average, representative, or typical respondent” from the perspective of respondent *i*. We will elaborate on that in the next section.

### Theoretical argument for peer-predicted choice

Assume that we have conducted a conjoint survey where each choice set was accompanied by two questions: the first question was about one’s personal choice within the set (i.e. own-choice question), while the second question was about the probability of how others would choose (i.e. peer-prediction question). While the first question provides data for the derivation of an individual’s personal utility, the second question allows us to infer the predicted utility structure. (We do not assume that the peer-predictions are a Bayesian posterior; instead we assume that they are simply the result of internal polling).

Let us consider a choice set *r* which consists of two alternatives {A,B}. Our respondent *i* reports their own choice, and their predictions for the percentage of people who will choose each of the alternatives. Respondents form this latter estimate on the basis of information from their social circle, in the way described by [1]: when people engage in predictions about their peers’ choices they in fact perform mental “polling” of their friends, acquaintances, family members etc. If a respondent’s social circle consists of *m* people, then they will mentally poll these *m* people.

By ${{\tilde{v}}_{rm}}^{i}$ we denote the peer-prediction that respondent *i* assigns to the alternative A in the choice set *r* on the basis of the social circle of size *m*. We define the associated peer-choice ${Y_{rm}}^{i}$ predicted by respondent *i* in the following way: ${Y_{rm}}^{i}\;=\;1$ if ${{\tilde{v}}_{rm}}^{i}>0.5;\;{Y_{rm}}^{i}\;=\;0$ if ${{\tilde{v}}_{rm}}^{i}<0.5$. For choice set *r* where ${{\tilde{v}}_{rm}}^{i}\;=\;0.5$, we consider that the respondent has no preference for either of the alternatives. We consider the respondent to be indecisive between the two alternatives. Therefore, we model that situation as if the respondent has chosen the no-choice option. Extant research [1, 2, 28] shows empirically that peer-choices formed on the basis of social sets result in better prediction of the winning alternative (i.e. the alternative chosen by the majority). If, for a choice set *r*, by *T*_*r*_ we denote the true choice made by the majority of the population, then this means that we can expect peer-choices to be better approximations of *T*_*r*_ than own-choices. The theoretical support for that is provided in the S1 Appendix. This is true even if we allow circle members’ choices to be dependent, as well as if we allow a certain amount of bias in respondents’ social circles.

Recall that collecting the peer-choices provided by the person *i* across all choice sets allows us to estimate the utilities for the typical respondent, as seen by the respondent *i*. We denote these utilities by vector ${\beta_{m}}^{i}$. Following the usual assumptions of the Hierarchical Bayes algorithm for conjoint estimation, the individual utilities ${\beta_{m}}^{i}$ associated with peer-choices are normally distributed with mean equal to the true utility $\overset{-}{\beta }$. In other words, ${\beta_{m}}^{i}\;=\;{{\overset{-}{\beta }}_{\;}}^{\;}+{\varepsilon_{m}}^{i}$, where ${\varepsilon_{m}}^{i}\sim N\left(0,W\right)$. The true utilities $\overset{-}{\beta }$ correspond to the true population choices *T*_*r*_, for all choice sets *r*. As peer-choices are expected to be a better approximation of the majority choices than own-choices, we would expect the utilities estimated from peer-choices to be closer to the real utilities ${\overset{-}{\beta }}_{\;}$.

To investigate if that is true, we conduct four experiments where we compare the realized shares of preferences with those predicted by the peer-choice model and with those predicted by the traditional own-choice model.

### Experiments

The experimental work in this study was approved by the Committee on the Use of Humans as Experimental Subjects of the Massachusetts Institute of Technology (protocol number E-1340). Subjects were shown a written consent statement before the start of the online experiment. They expressed their consent by clicking on the YES icon.

All experiments were conducted with the Mechanical Turk community. Subjects were based in the US, they expressed interest in the category, and they were screened to represent potential buyers. To achieve the latter, in all the experiments we excluded people who reported that they neither owned the product nor wanted to buy it in the future, as we judged them not to be in the market.

In Experiment 1 we used streaming media players. In Experiment 2 we repeated the same procedure one month later with a different group of people. In Experiment 3 we repeated the conjoint experiment on fitness/activity trackers. The first three experiments represent a within-subjects study, so we conducted a between-subjects study in Experiment 4 for additional validation. That last experiment was performed on smartwatches.

For all four experiments, the attributes and levels were sourced from the trade press and verified for comprehension with several product users in face-to-face interviews. We selected 5 attributes in the case of streaming media players, 8 attributes in the case of fitness/activity trackers, and 5 attributes in the case of smartwatches. Each attribute had at least two and at most 4 levels, making this a good case for the application of choice based conjoint analysis (Eggers and Sattler, 2011). Finished surveys were then pre-tested with a small group of potential users.

The first three experiments have a similar setup. Subjects saw twenty binary choice sets each. Fifteen of these sets were used to compute utilities, three were holdout sets reserved for validation, and two were repetitions of previously shown choice sets that were intended to screen for lack of attention. The three holdout sets were choice sets number 6, 12, and 18. The position of these sets was fixed for every respondent. Other choice sets were randomized. After holdout choice set 18, we repeated sets 6 and 18 to check for attention. Respondents who gave inconsistent answers across the two presentations of set 18 were deemed inattentive and excluded.

In Experiment 4 the survey was shorter, as it contained 15 binary choice sets in total compared to 20 in the first three experiments, and almost all non-conjoint questions were omitted. For that reason, we did not expect fatigue to be a big problem and we did not screen for attention. The main differing feature of Experiment 4 was that validation was performed on a separate sample consisting of different respondents.

We recorded the overall time that respondents spent on the survey, and we introduced a threshold below which we concluded that subjects could not give reliable answers. For the first three experiments that threshold is 300 seconds, and for the fourth experiment it is 150 seconds. This latter threshold is lower than in the previous three experiments because the survey was shorter and required less effort to complete.

After exclusion of subjects on all the criteria described above (not being in the market, time threshold, and checking for attention/fatigue), in Experiment 1 we retained 70% of the sample (56 subjects), in Experiment 2 we retained 68% of the sample (55 subjects), and in Experiment 3 we retained 83% of the sample (66 subjects). In Experiment 4 we used only the first two criteria (not being in the market and time threshold), which left us with 86.5% of the sample (225 subjects).

Each of the four experiments started with a short description of the testing procedure, explaining to subjects that they would be asked to make repeated choices between two alternatives. We also gave a very short description of the product (explaining what it was and how it was used). Each choice task consisted of the visual and text descriptions of the two offered alternatives followed by: (1) the question about which alternative the respondent would choose, and (2) the question that asked the respondent to make a peer-prediction. The exact wording of the second question was “What percentage of your peers would choose the same alternative as you?”, which was sufficient to infer the binary distribution across alternatives. As for possible order effects of these two questions, we relied on the doctoral thesis [29] which showed that the question order does not produce any significant effects.

Further details of methods and materials for experiments necessary to reproduce experiments (such as descriptions of choice sets) are provided in the S2 Appendix.

In all experiments we used the Bayesian Truth Serum (BTS) scoring rule invented in [30], which incentivizes truthful reporting. The two questions asked in each choice set (personal choice question plus peer prediction question) are also required for implementation of BTS, so in this way in each choice set respondents were incentivized to tell the truth. Subjects were told that BTS rewards truthfulness and the ability to predict what others would do, that we would compute the scores for each one of them based on their answers, and that the top 25% of people with the highest scores would receive an additional award consisting of 75% of the base payment. BTS has shown to be effective in incentivizing respondents in both laboratory [31] and online experiments [32, 33]. In Experiment 4 we also incentivized all the holdout questions in the validation sample with BTS.

The partial orthogonal design for the choice based conjoint analysis was produced by JMP Pro 14 SAS software. The same software was used for estimation of utilities via the Hierarchical Bayes model. A brief outline of the HB approach in conjoint is given earlier in this paper. SAS JMP employs a version of the Train algorithm that incorporates Adaptive Bayes and Metropolis-Hastings approaches. All Hierarchical Bayesian models were estimated with 300,000 iterations, 150,000 of them for burn-in. We allowed respondents to report any percentage between 0% and 100% as their peer-prediction. In those cases when respondents’ peer prediction was equal to 50%, we used the JMP “no-choice feature” which enters the respondent’s choice indicator as missing (this option is used if the respondent does not express a preference for any of the alternatives).

### Product attributes and further details

#### Experiment 1

The product chosen was a streaming media player, which can be plugged into any HDTV and connected to the existing WiFi network, thus enabling streaming of video and music content from online services. Respondents were informed that the profiles we showed them differed only in the following attributes: (1) price (40$, 50$, $70, $80), (2) resolution (1080, 4K), (3) remote control (point anywhere, point in sight, none), (4) ability to use the product in hotels, dorms, etc. (yes, no), and (5) inclusion of extra popular channels (Amazon+Direct TV, Amazon+FVG, FVG+Direct TV, all three).

#### Experiment 2

Experiment 2 used the same product and attributes as Experiment 1. It was performed about a month after the first one, with a different set of respondents. We added one extra holdout set that consisted of three real products to further test predictive ability.

As explained before, we used the same three holdout sets as in Experiment 1. The additional holdout set consisted of three real products that were available on the market at the time of testing: ROKU, Chromecast Ultra, and ROKU Premiere. All three products come in the form of USB sticks, and were the three most popular choices in that category and format at the time of testing.

#### Experiment 3

The third experiment used a fitness/activity tracker, a different type of product. While streaming media sticks are used by the majority of the population (as watching TV is a favorite pastime), the fitness tracker is aimed at fitness enthusiasts. The instructions made it clear that only respondents interested in fitness should participate in the MTurk task.

The fitness trackers that we tested were bracelets made of elastomer material. Each product automatically tracked steps, calories burned and distance travelled. Each synced wirelessly with users’ mobile phone and/or computer, using a free app to display the stats and activity data. All tested products had a sleep tracking system and rechargeable battery. The products were differentiated on the following attributes: (1) price ($79, $99, $129), (2) heart monitor included (yes, no), (3) GPS included (yes, no), (4) workout tracking in real time (yes, no), (4) water resistance (splash, swim), (5) display and notifications (calls text and calendar, calls and text, none), and (6) battery life (5 days, 7 days, 14 days). The setup of the experiment was the same as in the first two cases. There were three holdout sets, which were placed in the same positions as in the previous experiments (i.e. position 6, 12, and 18). Furthermore, as in Experiment 2, there was an additional holdout set containing three different real fitness trackers.

#### Experiment 4

For additional validity, we designed a between-subjects experiment. We recruited 260 respondents from the MTurk panel to perform a conjoint exercise. The product was a smartwatch, with differentiating attributes: product shape (rectangular digital, round classic, round sporty), brand (Apple, Samsung, Garmin), fitness tracking (yes, no, advanced), heart/health tracking (basic, standard, advanced), and price ($200, $300, $400). Fifteen binary choice sets were presented in random order.

A total of 225 subjects remained after exclusion of those who were not in the market and those who were considered inattentive due to too little time spent on the survey. They were randomly divided into two groups.

Further, we recruited a different group of 80 respondents a week after the first survey (we will refer to them as Group 3 or validation sample). After we excluded answers from subjects who said they did not own a smartwatch and they were not planning to buy it in the future, we were left with 73 respondents (we did not use any minimal time threshold as in the other experiments, because these questions required very little time). The validation sample subjects were showed eight sets of three real smartwatches chosen from the following products: Apple 2, Apple 3, Apple 4, Samsung Gear S3 Classic, Samsung Galaxy, and Garmin Fenix 5. To make the task easier and more salient, every photograph of a real product was accompanied with a list of attribute levels that were not visible from the picture (health/heart tracking, fitness tracking, brand and price), which were described the same way as in the first part of the experiment. In each 3-alternative choice set, subjects were asked to choose their preferred product and report the percentages of others who would choose each of the alternatives. Subjects were informed that their answers would be used to compute Bayesian Truth Serum scores, that the scores reward honest answers and knowledge of others, and that they would be rewarded according to their scores.

## Results

We use the data from the four experiments to test our theoretical argument that the peer-choice model provides a more accurate overall share of preference than the traditional approach. We test this by comparing prediction errors from both models (own-choice and peer-choice). Part-worths estimated from own-choice and part-worths estimated from peer-choice models are utilized to calculate the predicted share of alternatives.

We compute preference shares using average values of individual respondents’ part-worths. More precisely, if ${\beta_{l}}^{i}$ is respondent *i*’s part-worth for attribute/level *l*, then by *β*_*l*_ we denote the mean taken over all the respondents. In a two-alternative set *r* consisting of alternatives A and B, the preference share for alternative A is computed as

$$P\left(alternative\;A\;is\;chosen\right)\;=\;\frac{\text{exp}\;(\sum_{l}{{\beta_{l}}^{\;}\;}^{\;}{x_{rlA}}^{\;})}{\text{exp}\;\left(\sum_{l}{{\beta_{l}}^{\;}\;}^{\;}{x_{rlA}}^{\;}\right)+\text{exp}\;(\sum_{l}{{\beta_{l}}^{\;}\;}^{\;}{x_{rlB}}^{\;})},$$

where *x_rlA_* and *x_rlB_* are dummies designating levels of attributes present in the alternatives A and B. In a three-alternative set consisting of alternatives A, B and C, the preference share of A is

$$P\left(alternative\;A\;is\;chosen\right)\;=\;\frac{\text{exp}\;(\sum_{l}{{\beta_{l}}^{\;}\;}^{\;}{x_{rlA}}^{\;})}{\text{exp}\;\left(\sum_{l}{{\beta_{l}}^{\;}\;}^{\;}{x_{rlA}}^{\;}\right)+\text{exp}\;\left(\sum_{l}{{\beta_{l}}^{\;}\;}^{\;}{x_{rlB}}^{\;}\right)+\text{exp}\;\left(\sum_{l}{{\beta_{l}}^{\;}\;}^{\;}{x_{rlC}}^{\;}\right)}.$$

For estimation purposes we utilize 300,000 draws in the MCMC algorithm, out of which the last 150,000 are retained. Each draw produces a vector of individual part-worths (${\beta_{l}}^{i}$), and their associated averages (*β*_*l*_) For each draw, the latter are used to produce probabilities that alternatives are chosen (i.e. prediction shares). To arrive at the overall prediction for preference shares, we average these probabilities over the 150,000 draws.

Those estimates are calculated for both own-choice model and peer-choice model, and are compared with the actual share of choice for that alternative. We define error as the absolute value of the difference between the actual share of a given alternative (i.e. the percentage of respondents who actually chose the alternative) and the predicted share (i.e. the estimated probability that that alternative would be chosen). Then we examine ordered couples where the first coordinate is the error obtained from the own-choice model, while the second coordinate is the error obtained from the peer-choice model, and we use matched pairs test to check whether peer-choice errors are significantly smaller.

Practically, in a two-alternative choice set each alternative gives rise to such an ordered couple, so we use only the error associated with one of them as the other one is symmetric. In the three-alternative sets we report the ordered pairs for only two of the alternatives, since the third one is dependent on them.

### Overall share of preference: Experiments 1, 2, and 3

In these three within-subjects experiments there are three holdout sets (set 6, 12, and 18) which were set aside and not used for estimation of part-worths. In Experiment 2 and Experiment 3 there is also one additional set each containing three real products. We consider these eleven sets from the first three experiments together, and we compare prediction errors from both models (from both own-choice and peer-choice). This gives us 13 data points (because three-alternative sets provide two data points). As explained above, in the three-alternative choice sets we exclude the third alternative as redundant: these excluded alternatives are ROKU Premiere in Experiment 2, and Fitbit Charge in Experiment 3. The data is presented in Table 1.

**Table 1:** Prediction errors: The own-choice model and the peer-choice model (Experiments 1, 2, and 3).

	Choice sets	Predicted shares of preference		Realized shares of preference	Error own-choice ^a^	Error peer-choice ^b^
		Own-choice	Peer-choice			
Experiment 1	Holdout set 6	0.46*	0.53*	0.79	0.32	0.26
	Holdout set 12	1.00	0.96	0.79	0.21	0.17
	Holdout set 18	0.84	0.69	0.70	0.14	0.01
Experiment 2	Holdout set 6	1.00	0.79	0.77	0.23	0.02
	Holdout set 12	1.00	0.91	0.69	0.31	0.22
	Holdout set 18	0.93	0.78	0.75	0.18	0.03
	ROKU	0.97	0.79	0.46	0.51	0.33
	Chromecast ultra	0.00	0.04	0.23	0.23	0.19
Experiment 3	Holdout set 6	0.08	0.18	0.29	0.21	0.11
	Holdout set 12	0.65	0.53	0.45	0.20	0.08
	Holdout set 18	0.98	0.90	0.89	0.09	0.01
	Fitbit Alpha	0.00	0.03	0.04	0.04	0.01
	Fitbit Flex	0.00	0.01	0.10	0.10	0.09

^a^ Error own-choice is defined as absolute value of the difference between shares predicted from the own-choice model and the realized shares from the validation sample.

^b^ Error peer-choice is defined as absolute value of the difference between shares predicted from the peer-choice model and the realized shares from the validation sample.

* Standard errors for all means in both models are smaller than 0.001.

**Fig 1:**
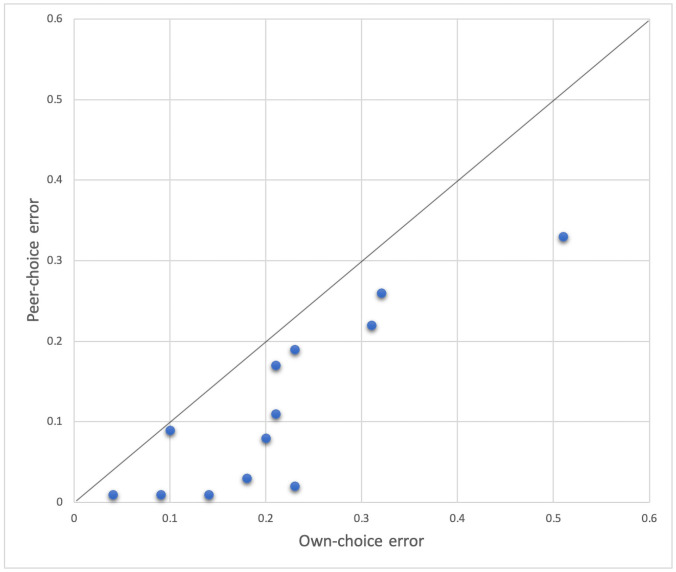
Comparison of the two matched errors: Peer-choice and own-choice in Experiments 1, 2, and 3.

We obtain that the average error from the peer-choice model is 0.12, while the average error from the own-choice model is 0.21. The Wilcoxon signed rank test is significant with p = 0.0002 for the two-tailed test. Fig 1 shows the relationship between the error from the own-choice model and the peer-choice model (the line in the graph is the diagonal). It is easy to see that almost all the points are below the diagonal, which means that in the large majority of cases the error from the own-choice model is larger than the error from the peer-predicted model.

### Overall share of preference: Experiment 4

The study was performed on a sample of 225 subjects (after data cleaning), which was randomly divided into two equal groups. We computed both own-choice part-worths and peer-choice part-worths for both groups. First we used own-choice part-worths from Group 1 and peer-choice part-worths from Group 2 to compute overall share predictions for the eight holdout sets consisting of real products. Both were compared to the realized shares from the third independent group (the validation sample), which was shown only the eight holdout sets. After that, Groups 1 and 2 were switched and the same procedure was repeated, so that Group 1 was used for computation of peer-choice predictions, while Group 2 was used for calculation of own-choice predictions (Table 2).

**Table 2:** Prediction errors: The own-choice model and the peer-choice model (experiment 4).

	Choice sets	Product in the choice set	Predicted shares of preference		Realized shares of preference	Error own-choice ^a^	Error peer-choice ^b^
			Own-choice	Peer-choice	Validation sets		
Group 1 is used for estimation of own-choice utilities Group 2 is used for estimation of peer-choice utilities Group 3 is the validation sample	Set 1	Apple 2	0.62*	0.60*	0.24	0.38	0.36
		Apple 3	0.38	0.38	0.69	0.31	0.31
		Samsung Gear S3 Classic					
	Set 2	Apple 4	0.55	0.62	0.63	0.08	0.01
		Samsung Gear S3 Classic	0.37	0.25	0.26	0.11	0.01
		Garmin Fenix 5					
	Set 3	Apple 3	0.68	0.45	0.22	0.46	0.23
		Apple 4	0.17	0.39	0.57	0.4	0.18
		Samsung Gear S3 Classic					
	Set 4	Samsung Galaxy Watch	0.81	0.61	0.61	0.2	0
		Samsung Gear S3 Classic	0.01	0.10	0.07	0.06	0.03
		Garmin Fenix 5					
	Set 5	Apple 3	0.84	0.71	0.71	0.13	0
		Samsung Galaxy Watch	0.16	0.25	0.24	0.08	0.01
		Samsung Gear S3 Classic					
	Set 6	Apple 3	0.95	0.82	0.72	0.23	0.1
		Samsung Gear S3 Classic	0.002	0.05	0.08	0.078	0.03
		Garmin Fenix 5					
	Set 7	Apple 3	0.75	0.49	0.21	0.54	0.28
		Apple 4	0.21	0.42	0.61	0.4	0.19
		Garmin Fenix 5					
	Set 8	Apple 4	0.86	0.79	0.74	0.12	0.05
		Samsung Gear S3 Classic	0.005	0.05	0.13	0.125	0.08
		Garmin Fenix 5					
Group 1 is used for estimation of peer-choice utilities Group 2 is used for estimation of own-choice utilities Group 3 is the validation sample	Set 1	Apple 2	0.66	0.53	0.21	0.45	0.32
		Apple 3	0.34	0.45	0.61	0.27	0.16
		Samsung Gear S3 Classic					
	Set 2	Apple 4	0.63	0.54	0.63	0	0.09
		Samsung Gear S3 Classic	0.27	0.33	0.26	0.01	0.07
		Garmin Fenix 5					
	Set 3	Apple 3	0.41	0.48	0.22	0.19	0.26
		Apple 4	0.41	0.32	0.57	0.16	0.25
		Samsung Gear S3 Classic					
	Set 4	Samsung Galaxy Watch	0.73	0.68	0.61	0.12	0.07
		Samsung Gear S3 Classic	0.01	0.09	0.07	0.06	0.02
		Garmin Fenix 5					
	Set 5	Apple 3	0.7	0.68	0.71	0.01	0.03
		Samsung Galaxy Watch	0.3	0.28	0.24	0.06	0.04
		Samsung Gear S3 Classic					
	Set 6	Apple 3	0.86	0.83	0.72	0.14	0.11
		Samsung Gear S3 Classic	0.003	0.05	0.08	0.077	0.03
		Garmin Fenix 5					
	Set 7	Apple 3	0.47	0.55	0.21	0.26	0.34
		Apple 4	0.46	0.37	0.61	0.15	0.24
		Garmin Fenix 5					
	Set 8	Apple 4	0.86	0.76	0.74	0.12	0.02
		Samsung Gear S3 Classic	0.004	0.06	0.13	0.126	0.07
		Garmin Fenix 5					

^a^ Error own-choice is defined as absolute value of the difference between shares predicted from the own-choice model and the realized shares from the validation sample.

^b^ Error peer-choice is defined as absolute value of the difference between shares predicted from the peer-choice model and the realized shares from the validation sample.

* Standard errors for all means in both models are smaller than 0.001.

**Fig 2:**
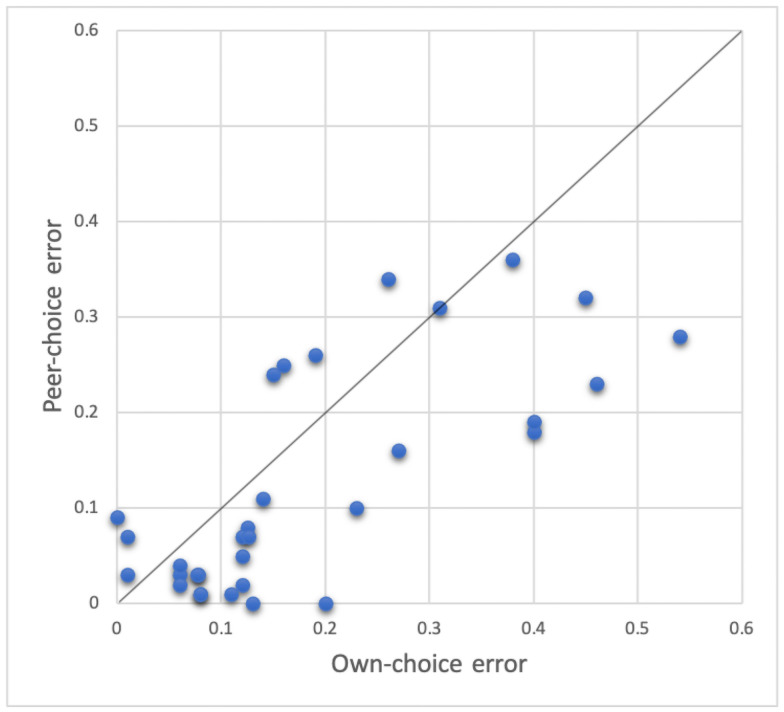
Comparison of the two matched errors: Peer-choice and own-choice in Experiment 4.

We computed absolute errors associated with own-choice predictions and with peer-choice predictions. We pooled the absolute errors from both computations and applied matched pairs t-test and Wilcoxon signed rank test, which were highly significant with p = 0.001 and p = 0.003 for the two-tailed test, respectively. The pairs of own and peer errors are presented in Fig 2. As before, it is clear that the majority of points are below the diagonal.

In summary, data from all four experiments shows that statistically the peer-choice model produces a significantly smaller error compared to the own-choice model.

Having said that, as this is the first study on the topic of peer-choice conjoint, it has limitations that will be resolved by future research. One limitation of our dataset is that realized shares that are used as a validation benchmark happen to be statistically closer to a chance model (i.e., 0.5 in the binary choices and 0.33 in the three-alternative holdout sets), compared with the own-choice predictions. Consequently, as the peer-choice model better approximates real choices, peer-choice predictions are also closer to chance compared to own-choice predictions. However, it is known that if respondents mistakenly choose alternatives that do not represent their true utility, the magnitude of their part-worths will decrease, and their choice predictions for different alternatives will likely be similar [34]. Although we believe that the closeness to chance in our case is the result of improvement in prediction and not of noise introduction, this question can be best resolved by conducting new studies on different datasets.

## Conclusion

We propose a new method of eliciting market research information, based on asking respondents to predict the choices of their peers. Peer predictions expand the virtual sample size, and tap individuals’ knowledge of their local crowd preferences.

We focus on conjoint analysis to illustrate the viability of the new approach. Although there has been much work on the estimation in conjoint analysis, up until now the main paradigm was to elicit respondents’ personal choice as a source of preference structure. With this paper we open up another perspective by proposing to consider respondents’ opinion of what others would choose, instead of eliciting their personal choice.

First, we develop a theoretical argument that shows that relying on peer-knowledge yields improvements: namely, larger social circles allow for more accurate utility estimates and for better predictions of preference share. Our theoretical model was confirmed in four online experiments: three experiments performed validation within subjects and the fourth one validated the method between subjects.

In engaging respondents’ meta-knowledge, our approach is similar in spirit to Security Trading of Concepts (STOC) by [23], where participants use their knowledge about others’ preferences in a prediction market. While we stay methodologically within the framework of conjoint analysis, like STOC our method predicts the overall share of preference and can also be used for choosing the most attractive concepts for the overall market.

We can envision some additional benefits that the new approach may deliver. First, by drawing on people’s social circles we collect information about a larger number of people, so in a way we involve a larger number of subjects. Another advantage of the peer-choice model is that it is particularly helpful when people are reluctant to report personal choices that may reveal potentially sensitive private information or preferences.

Our method can be combined with the traditional conjoint approach. Our approach does not preclude the use of the personal choice question: instead we advocate the addition of peer-choice to the personal choice question. An additional advantage is that those two questions together allow for easy survey incentivization through the use of Bayesian Truth Serum.

Although larger circles should lead to better prediction, the accuracy is moderated by the level of choice dependence within the social circles: information increases as social circle members become mutually more independent. Smaller independent circles can impart as much information as larger circles where members are more dependent in their choices. Our model should perform best in situations where social circles are large and diverse. Future research may investigate if the model accuracy can be improved by screening for respondents with large and diverse circles.

## Supporting information

S1 Appendix More detailed theoretical argument. (DOCX)

S2 Appendix Details of the conjoint study. (DOCX)

S1 Data Experiment 1 data. (XLSX)

S2 Data Experiment 2 data. (XLSX)

S3 Data Experiment 3 data. (XLSX)

S4 Data Experiment 4 data. (XLSX)

S1 Questionnaire Experiment 1 questionnaire no images. (PDF)

S2 Questionnaire Experiment 2 questionnaire no images. (PDF)

S3 Questionnaire Experiment 3 questionnaire no images. (PDF)

S4 Questionnaire Experiment 4 questionnaire no images. (PDF)

S5 Questionnaire Experiment 4 questionnaire real sets no images. (PDF)

S1 Code (DOCX)
