# Supplementary material for: Predicted preference conjoint analysis
Source: PLoS One. 2021 Aug 26;16(8):e0256010. doi: 10.1371/journal.pone.0256010 (PMC8389521; doi:10.1371/journal.pone.0256010)
Supplement: S1 Appendix — (DOCX) [file pone.0256010.s001.docx]

**S1 Appendix: More detailed theoretical argument**

We assume that when people engage in predictions about their peers’ choices, they in fact perform mental “polling” of their friends, acquaintances, family members etc. If their social circle consists of *m* people, then they mentally poll these *m* people. We assume that the choices of the members of the social circle are distributed as a Bernoulli distribution with mean equal to the probability of choosing one of the alternatives, say the alternative A, a respondent *k* will report peer-prediction ${\tilde{v}_{m}}^{k}=\frac{X_{1}+X_{2}+\ldots+X_{m}}{m}$, where the circle members are represented by Bernoulli variables $X_{i}$ (these are actually ${X_{i}}^{k}$, but here we drop *k* for simplicity). These variables $X_{i}$ do not need to be identically distributed. Since our discourse in this appendix is developed for one choice set and is easily replicable generally, for simplicity of notation we drop index *r* that denotes a choice set.

We allow the variables$X_{i}$ to be dependent. As one possible measure of dependence in the social circle we use “average covariance” among circle members, which we define as $2\frac{1}{m^{2}-m}\sum_{i<j} Cov\left( X_{i},X_{j} \right).$ It is reasonable to expect that on average people in large networks are less interdependent than those in small networks, resulting in decreasing level of average covariance between circle members. We assume that this decrease follows the function $m^{-\delta}$, where $\delta>0$. The function $m^{-\delta}$ is often used in marketing modeling, and with different choices of $\delta$ it can accommodate both fast and slow descent.

It can be shown that, with this assumption, individuals’ peer-predictions approach the true share of preference in the population, and this prediction becomes more precise when circles become larger. We first show that as the social circle increases, the variance of the prediction $\frac{X_{1}+X_{2}+\ldots+X_{m}}{m}$ becomes smaller. This can be formally stated as Proposition 1.

***Proposition 1.***

*If* $2\frac{1}{m^{2}-m}\sum_{i<j} Cov\left( X_{i},X_{j} \right)<Km^{-\delta}$*, then* $\lim_{n\to\infty} Var\left( \frac{X_{1}+X_{2}+\ldots+X_{m}}{m} \right)=0.$

Proof:

We measure that average dependence among members of the social circle using $2\frac{1}{m^{2}-m}\sum_{i<j} Cov\left( X_{i},X_{j} \right)$, we assume that it decreases with the size of the circle, and we assume that this decrease follows the function $m^{-\delta}.$ Mathematically this means that there exists $0<\delta$such that for every *m* large enough, it holds that $2\frac{1}{m^{2}-m}\sum_{i<j} Cov\left( X_{i},X_{j} \right)<Km^{-\delta}$, where *K* is a constant independent of *m*. Let $X_{1}, X_{2},\ldots,X_{m}$ be Bernoulli variables such that $X_{i}\sim\left( \begin{matrix} 0 & 1 \\ 1-{v_{i}} & v_{i} \end{matrix} \right)$*,* and let $R_{m}=(X_{1}+X_{2}+\ldots+X_{m})$*.*

Then $2\frac{1}{m^{2}}\sum_{i<j} Cov\left( X_{i},X_{j} \right)<2\frac{1}{m^{2}-m}\sum_{i<j} Cov\left( X_{i},X_{j} \right)<Km^{-\delta}.$ Notice that

$$Var\left( \frac{R_{m}}{m} \right)=Var\left( \frac{1}{m}\sum_{i=1}^{m} X_{i} \right)=\frac{1}{m^{2}}Var\left( \sum_{i=1}^{m} X_{i} \right)=\frac{1}{m^{2}}\sum_{i=1}^{m} \sum_{j=1}^{m} Cov(X_{i},X_{j} )$$

$$=\frac{1}{m^{2}}\sum_{i=1}^{m} Var(X_{i})+2\frac{1}{m^{2}}\sum_{i<j} Cov\left( X_{i},X_{j} \right)=$$

$$=\frac{1}{m^{2}}\sum_{i=1}^{m} v_{i}\left( 1-{v_{i}} \right)+2\frac{1}{m^{2}}\sum_{i<j} Cov\left( X_{i},X_{j} \right)=$$

$$<\frac{1}{m^{2}}\sum_{i=1}^{m} 1+2\frac{1}{m^{2}}\sum_{i<j} Cov\left( X_{i},X_{j} \right)<\frac{1}{m}+ Km^{-\delta}$$

Because of the assumption involving $\delta,$ we have that $Var\left( \frac{R_{m}}{m} \right)< \frac{1}{m}+Km^{-\delta}$. This converges to zero as *m* tends to infinity, thus $\lim_{m\to\infty} Var\left( \frac{R_{m}}{m} \right)=0.$

Q.E.D.

Next we show that the predicted probability of choosing alternative A approaches the true probability of choosing A when *m* increases. We use the above assumption that average covariance among circle members decreases with the circle size. We also assume that as circle increases it becomes more representative of the entire population. More precisely, if random variables $X_{i}$which describe individual circle members are Bernoulli distributed with parameters $v_{i},$we assume that $\frac{1}{m}\sum_{i=}^{m} v_{i}\to v$. Then the peer prediction approaches the true probability $v$*.*

***Proposition 2.***

*Let*$\boldsymbol{v}$*denote the true share of chosen alternative in the population. If* $2\frac{1}{m^{2}-m}\sum_{i<j} Cov\left( X_{i},X_{j} \right)<Km^{-\delta}$ *and* $\frac{1}{m}\sum_{i=}^{m} v_{i}\to v$*, then* $\frac{\boldsymbol{X}_{\boldsymbol{1}}\boldsymbol{+}\boldsymbol{X}_{\boldsymbol{2}}\boldsymbol{+\ldots+}\boldsymbol{X}_{\boldsymbol{m}}}{\boldsymbol{m}}\underset{\to}{\boldsymbol{P}}\boldsymbol{v}$***.***

Proof:

We first define $H_{m}=\frac{R_{m}}{m}-\frac{1}{m}\sum_{i=}^{m} v_{i}$. By Proposition 1 we have that $\lim_{m\to\infty} Var\left( \frac{R_{m}}{m} \right)=0.$ Now we can use Tchebychev inequality, which shows that as *m* increases,

$$P\left( \left| H_{m} \right|>\varepsilon\right)\leq\frac{Var\left( H_{m} \right)}{\varepsilon^{2}}=\frac{Var\left( \frac{R_{m}}{m} \right)}{\varepsilon^{2}}\to0.$$

This means that as *m* increases, $H_{m}\underset{\to}{P}0$, and then ${\frac{R_{m}}{m}=H}_{m}+\frac{1}{m}\sum_{i=}^{m} v_{i}\underset{\to}{P}v$

Q.E.D.

Now we discuss the situation where the assumption $\frac{1}{m}\sum_{i=}^{m} v_{i}\to v is not satisfied.$ This happens when social circles are biased, i.e. when they do not get more representative of the population as they get larger.

We show that peer choice approach can be helpful even when social circles are biased. Assume that there are two segments in the population, $S_{1}$ and $S_{2},$ and that people are identically distributed within each segment. The probability of belonging to the segment $S_{l}$is denoted by $s_{l}.$The choices of people who are in segment $S_{l}$can be described by Bernoulli random variables $X_{l}\sim\left( \begin{matrix} 0 & 1 \\ 1-{v_{l}} & v_{l} \end{matrix} \right)$.

Let ${m_{l}}^{i}$ denote the number of people from segment $S_{l}$ that are in respondent’s *i* social circle. Then $E\left( \frac{R_{m}}{m} \right)=\frac{{m_{1}}^{i}}{m}v_{1}+\frac{{m_{2}}^{i}}{m}v_{2}$ (recall that $R_{m}=(X_{1}+X_{2}+\ldots+X_{m})$).

Let us assume that the respondent *i* belongs to segment $S_{1}$ (for simplicity now we drop index *i*). If respondent *i* prefers to socialize with people from their own segment, than as *m* increases $\frac{m_{1}}{m}\to s_{1}+\theta$, while $\frac{m_{2}}{m}\to s_{2}-\theta$, where $1>\theta>0$ is the measure of the bias. Then respondent *i’*s expected prediction will converge to

${E\left( \frac{R_{m}}{m} \right)\to s}_{1}v_{1}+s_{2}v_{2}+\theta v_{1}-\theta v_{2}=v+\theta(v_{1}-v_{2}) (*)$

If the social circle of respondent *i* is representative of the population, then $\theta=0$ and the peer prediction is accurate. However, the above expression is different from $v$ if $\theta\neq0$. Recall that peer choice $Y_{m}$is defined as 1 if $\frac{R_{m}}{m}>0.5,$and 0 if $\frac{R_{m}}{m}<0.5$ (in case $\frac{R_{m}}{m}=0.5$, peer choice is undetermined).

If the social circle of respondent *i* does not include anyone from segment $S_{2}$, their expected prediction of peer probability to choose A is equal to the probability of their own choice for A, which is $v_{1}$. But if respondent *i* has even a small number of people from $S_{2}$ in their circle, that can improve their prediction of peer choice.

For example, assume that $v_{1}<0.5<v.$If respondent’s social circle would contain only people from their own segment $S_{1}$, then expected peer choice would be alternative B. It is easy to see that (*) can be rewritten as $E\left( \frac{R_{m}}{m} \right){\to v}_{1}+{(s_{2}-\theta)}(v_{2}-v_{1}).$If there is a group of circle members from segment $S_{2}$ who prefer option A, then observation of their choices may “nudge” the respondent *i*’s expected peer prediction over 0.5, so that it becomes closer to $v$, because ${v_{1}<0.5<v}_{1}+{(s_{2}-\theta)}(v_{2}-v_{1}).$ That would mean that the expected peer choice $Y_{m}$would become A, which is the expected choice of the population. Similarly, we can see that when $v_{1}>0.5>v,$ considering even a smaller-than-representative group of circle members from the other segment can push the expected peer prediction under the 0.5 threshold and thus closer to the true choice. This shows that we can have some degree of bias in respondents’ social circles and still produce more accurate results by peer choice method. Of course, if social circles are extremely polarized in the sense that for all respondents, they never include people from other segments, regardless of how large they get, then peer choice approach would not produce any improvements over traditional conjoint method.
