# Supplementary material for: Predicted preference conjoint analysis
Source: PLoS One. 2021 Aug 26;16(8):e0256010. doi: 10.1371/journal.pone.0256010 (PMC8389521; doi:10.1371/journal.pone.0256010)
Supplement: S2 Appendix — (DOCX) [file pone.0256010.s002.docx]

**S2 Appendix: Details of the conjoint study**

The experimental work in this study was approved by Committee on the Use of Humans as Experimental Subjects of the Massachusetts institute of Technology (protocol number E-1340). Subjects were presented with written consent before the start of the experiment.

**Experiments 1, 2 and 3 - study procedure:**

The study subjects were recruited from MTurk, and web-based conjoint questionnaire was administered by Qualtrics. Subjects saw twenty binary choice sets each. Fifteen of these sets were used to compute utilities, and three were holdout sets. Out of those three holdout sets, two sets were repeated to test for consistency and accuracy. The three holdout sets were choice set number 6, 12, and 18. The position of these sets were fixed for every respondent. The choice sets 1-5 were randomized, as well as choice sets 7-11 and choice sets 13-17. After the holdout choice set 18, we repeated the sets 6 and 18 to check for consistency. Since the same choice set was repeated so soon, and since the set in-between was already seen by subjects and as such did not require too much mental exertion, one can argue that those who paid attention to the survey should have given the same answer to set 18 both times. So the lack of consistent answers to the question in set 18 is used to identify respondents who were not paying sufficient attention, and hence these respondents were excluded. For easier understanding, the order of the sets is presented in the figure 1. Notice that every respondent saw the same choice sets at positions 6,12, 18, 19 and 20, regardless of what else they saw.

Randomized sets 1 to 5

Holdout set 6

Randomized sets 7 to 11

Hold-out set 12

Randomized sets 13 to 17

Hold-out set 18

Hold-out set 6

repeat

Hold-out set 18

repeat

Figure 1. The order of choice sets

Each experiment started with a short description of the testing procedure, explaining to subjects that they would be asked to make repeated choices between two alternatives. We also gave them a very short description of the product (explaining what it was and how it was used), considering that the tested products were rather novel at the time of testing. In the experiment each choice task consisted of the visual and text descriptions of the two offered alternatives followed by: (1) the question about which alternative the respondent would choose, and (2) the question that asked the respondent to make a peer-prediction. The exact wording of the second question was “What percentage of your peers would choose the same alternative as you?”, which was sufficient to infer the binary distribution across alternatives.

We recorded the overall time that respondents spent on the survey. Subjects who finished survey in less than 300 seconds were excluded. We also asked subjects if they owned a product, and if they were planning to buy it. Those who answered negative to both questions were considered not in the market and thus were excluded.

Experiment 1 and 2 involved a streaming media player which differed only in the following attributes: (1) price (40$, 50$, $70, $80), (2) resolution (1080, 4K), (3) remote control (point anywhere, point in sight, none), (4) ability to use the product in hotels, dorms, etc. (Yes, No), and (5) inclusion of extra popular channels (Amazon+Direct TV, Amazon+FVG, FVG+Direct TV, all three). In that last choice set, subjects were asked to choose the most preferred alternative. To make the task easier and more salient, every photograph of a real product was accompanied with a list of attribute levels that were not visible from the picture, which were described using the same way as in the first part of the experiment.

S1 Table presents 18 choice sets: 6, 12 and 18 were holdout sets, and the rest were used for utility estimation.

S1 Table. Experiment 1 and 2, choice sets

| Choice Set | Price | Resolution | Remote | Use in hotels | Extra channels |
| --- | --- | --- | --- | --- | --- |
| 1 | 50 | 4K | point of sight | yes | Amazon +DIRECTV |
| 1 | 40 | 4K | point anywhere | yes | AMAZON+FVG |
| 2 | 40 | 1080 | point of sight | no | Amazon +DIRECTV+ FVG |
| 2 | 50 | 4K | none | yes | FVG+DIRECTV |
| 3 | 50 | 4K | point anywhere | yes | FVG+DIRECTV |
| 3 | 80 | 4K | point of sight | yes | Amazon +DIRECTV |
| 4 | 70 | 1080 | none | no | AMAZON+FVG |
| 4 | 40 | 4K | point of sight | yes | FVG+DIRECTV |
| 5 | 80 | 1080 | point anywhere | no | Amazon +DIRECTV |
| 5 | 50 | 4K | point of sight | yes | AMAZON+FVG |
| 6 | 40 | 4K | point anywhere | yes | Amazon +DIRECTV |
| 6 | 50 | 1080 | point of sight | no | FVG+DIRECTV |
| 7 | 40 | 4K | point of sight | no | FVG+DIRECTV |
| 7 | 50 | 1080 | point anywhere | yes | Amazon +DIRECTV+ FVG |
| 8 | 50 | 1080 | point anywhere | yes | AMAZON+FVG |
| 8 | 80 | 4K | none | yes | FVG+DIRECTV |
| 9 | 50 | 4K | point of sight | no | AMAZON+FVG |
| 9 | 70 | 1080 | point anywhere | yes | FVG+DIRECTV |
| 10 | 80 | 1080 | point of sight | yes | FVG+DIRECTV |
| 10 | 70 | 4K | none | no | Amazon +DIRECTV |
| 11 | 70 | 1080 | point of sight | no | AMAZON+FVG |
| 11 | 50 | 1080 | point anywhere | no | Amazon +DIRECTV |
| 12 | 80 | 4K | point anywhere | no | FVG+DIRECTV |
| 12 | 70 | 1080 | none | yes | Amazon +DIRECTV |
| 13 | 80 | 4K | point anywhere | yes | AMAZON+FVG |
| 13 | 40 | 1080 | none | no | Amazon +DIRECTV+ FVG |
| 14 | 40 | 1080 | point of sight | yes | Amazon +DIRECTV+ FVG |
| 14 | 70 | 4K | point anywhere | no | FVG+DIRECTV |
| 15 | 40 | 4K | none | no | Amazon +DIRECTV+ FVG |
| 15 | 70 | 1080 | point of sight | no | Amazon +DIRECTV |
| 16 | 40 | 4K | point anywhere | no | Amazon +DIRECTV |
| 16 | 70 | 1080 | none | yes | FVG+DIRECTV |
| 17 | 70 | 4K | point anywhere | no | Amazon +DIRECTV+ FVG |
| 17 | 40 | 1080 | point of sight | yes | Amazon +DIRECTV |
| 18 | 70 | 1080 | point of sight | yes | FVG+DIRECTV |
| 18 | 80 | 1080 | none | yes | AMAZON+FVG |

Experiments 2 also contained one more holdout set consisting of three real-life product alternatives. These were ROKU, Chromecast Ultra, and ROKU Premiere.

Experiment 3 used a fitness/activity tracker. The products were differentiated on the following attributes: (1) price ($79, $99, $129), (2) heart monitor included (Yes, No), (3) GPS included (Yes, No), (4) workout tracking in real time (Yes, No), (4) water resistance (Splash, Swim), (5) Display and notifications: (Calls, text, and calendar, Calls and text, None), and (6) battery life (5 days, 7 days, 14 days). An additional holdout set contains three real prpoducts, these were Fitbit Alpha, Fitbit Flex, and Fitbit Charge. In that last choice set, subjects were asked to choose the one product they preferred the most. As in Experiments 1 and 2, photographs of those real product were described using the list of attribute levels that were used in the first part of the experiment. S2 Table presents 18 choice sets: 6, 12 and 18 were holdout sets, and the rest were used for utility estimation.

S2 Table. Experiment 3, choice sets

| Choice Set | Price | Heart rate | GPS | Workout tracking | Water resistance | Display and notifications | Battery life |
| --- | --- | --- | --- | --- | --- | --- | --- |
| 1 | 99 | yes | no | no | splash | OLED+call text+callendar | 14 days |
| 1 | 129 | no | yes | yes | swim | OLED +call text | 7 days |
| 2 | 99 | no | yes | no | swim | OLED+call text+callendar | 5 days |
| 2 | 129 | yes | no | yes | splash | OLED +call text | 7 days |
| 3 | 129 | no | no | no | swim | OLED+call text+callendar | 5 days |
| 3 | 99 | yes | yes | yes | splash | no display | 7 days |
| 4 | 79 | yes | no | no | swim | OLED +call text | 5 days |
| 4 | 99 | yes | yes | yes | swim | OLED+call text+callendar | 14 days |
| 5 | 79 | no | no | yes | swim | OLED+call text+callendar | 7 days |
| 5 | 129 | yes | yes | no | splash | OLED +call text | 14 days |
| 6 | 99 | yes | no | no | swim | no display | 7 days |
| 6 | 79 | no | yes | yes | splash | OLED +call text | 5 days |
| 7 | 129 | yes | yes | no | swim | OLED +call text | 7 days |
| 7 | 99 | no | no | yes | splash | no display | 14 days |
| 8 | 79 | no | yes | no | splash | OLED+call text+callendar | 7 days |
| 8 | 129 | yes | no | yes | swim | OLED +call text | 5 days |
| 9 | 129 | yes | no | no | splash | OLED+call text+callendar | 7 days |
| 9 | 99 | no | yes | yes | swim | OLED +call text | 14 days |
| 10 | 99 | yes | no | yes | swim | OLED +call text | 7 days |
| 10 | 129 | no | yes | no | splash | no display | 5 days |
| 11 | 99 | no | no | no | swim | OLED +call text | 14 days |
| 11 | 129 | yes | yes | yes | splash | no display | 7 days |
| 12 | 79 | no | no | yes | swim | OLED+call text+callendar | 5 days |
| 12 | 79 | yes | yes | no | splash | no display | 5 days |
| 13 | 99 | yes | yes | yes | splash | OLED +call text | 5 days |
| 13 | 99 | no | no | no | swim | no display | 7 days |
| 14 | 99 | no | yes | no | swim | OLED +call text | 7 days |
| 14 | 79 | yes | no | yes | swim | no display | 5 days |
| 15 | 99 | yes | yes | no | swim | OLED +call text | 14 days |
| 15 | 129 | no | no | yes | splash | OLED+call text+callendar | 7 days |
| 16 | 99 | yes | yes | no | splash | OLED +call text | 7 days |
| 16 | 79 | yes | yes | no | splash | no display | 5 days |
| 17 | 129 | no | no | yes | splash | OLED +call text | 14 days |
| 17 | 79 | yes | yes | no | swim | no display | 7 days |
| 18 | 99 | yes | no | yes | splash | OLED +call text | 5 days |
| 18 | 79 | yes | no | no | splash | no display | 5 days |

**Experiment 4 - study procedure:**

In this study the product was a smartwatch, with differentiating attributes: product shape (rectangular digital, round classic and round sporty), brand (Apple, Samsung, Garmin), fitness tracking (yes, no, advanced), heart/health tracking (basic, standard, advanced), and price ($200, $300, $400). 260 subjects were recruited from MTurk: we excluded those subjects who spent less than 180 seconds on the entire questionnaire, as well as those who reported not owning the product and not planning to buy it in the future. The web-based conjoint questionnaire was administered by Qualtrics. Subjects saw fifteen binary choice sets each, all of which were used to compute utilities. S3 Table presents 15 choice sets shown in the experiment.

S3 Table. Experiment 4 – choice sets

| Choice Set | Price | Shape | Fitness tracking | Health/heart monitoring | Brand |
| --- | --- | --- | --- | --- | --- |
| 1 | 400 | Rectangular | Advanced | Basic | Samsung |
| 1 | 300 | Round sporty | Yes | Standard | Apple |
| 2 | 200 | Round sporty | No | Standard | Apple |
| 2 | 300 | Rectangular | Yes | Advanced | Samsung |
| 3 | 200 | Round sporty | Yes | Basic | Apple |
| 3 | 300 | Round sporty | No | Standard | Samsung |
| 4 | 400 | Rectangular | No | Standard | Apple |
| 4 | 300 | Round sporty | Yes | Basic | Samsung |
| 5 | 200 | Round classic | Yes | Standard | Garmin |
| 5 | 300 | Rectangular | No | Basic | Samsung |
| 6 | 200 | Round classic | No | Standard | Samsung |
| 6 | 300 | Round sporty | Advanced | Standard | Apple |
| 7 | 200 | Round classic | No | Basic | Garmin |
| 7 | 300 | Rectangular | Yes | Standard | Apple |
| 8 | 300 | Round classic | No | Basic | Apple |
| 8 | 200 | Rectangular | Yes | Standard | Samsung |
| 9 | 400 | Round sporty | No | Standard | Samsung |
| 9 | 300 | Round classic | Advanced | Advanced | Apple |
| 10 | 300 | Rectangular | Advanced | Standard | Garmin |
| 10 | 400 | Round sporty | Yes | Advanced | Samsung |
| 11 | 400 | Round sporty | Advanced | Advanced | Garmin |
| 11 | 300 | Round classic | Yes | Basic | Apple |
| 12 | 200 | Round classic | No | Advanced | Samsung |
| 12 | 400 | Rectangular | Yes | Basic | Garmin |
| 13 | 400 | Round classic | Advanced | Standard | Samsung |
| 13 | 300 | Rectangular | No | Basic | Garmin |
| 14 | 200 | Rectangular | No | Advanced | Apple |
| 14 | 300 | Round classic | Yes | Standard | Samsung |
| 15 | 200 | Rectangular | Advanced | Standard | Samsung |
| 15 | 400 | Round classic | Yes | Advanced | Garmin |

Further, we recruited a different group of 80 respondents as a validation sample. As the questionnaire was very short, we did not use any minimal time threshold as in other experiments. We excluded subjects who said they did not own a smartwatch and they were not planning to buy it in the future. The validation sample subjects were showed eight sets of three real smartwatches chosen from the following product: Apple 2, Apple 3, Apple 4, Samsung Gear S3 Classic, Samsung Galaxy, and Garmin Fenix 5. To make the task easier and more salient, every photograph of a real product was accompanied with a list of attribute levels that were not visible from the picture (health/heart tracking, fitness tracking, brand and price), which were described using the same way as in the first part of the experiment. In each 3-alternative choice set subjects were asked to choose their preferred product, and report percentages of others who would choose each of the alternatives.

S4 Table: Experiment 4 validation sets

| Set | Product A | Product B | Product C |
| --- | --- | --- | --- |
| 1 | Apple 2 | Apple 3 | Samsung Gear S3 Classic |
| 2 | Apple 4 | Samsung Galaxy | Garmin Fenix |
| 3 | Apple 3 | Apple 4 | Samsung Galaxy |
| 4 | Samsung Galaxy | Samsung Gear S3 Classic | Garmin Fenix |
| 5 | Apple 3 | Samsung Galaxy | Samsung Gear S3 Classic |
| 6 | Apple 3 | Samsung Gear S3 Classic | Garmin Fenix |
| 7 | Apple 3 | Apple 4 | Garmin Fenix |
| 8 | Apple 4 | Samsung Gear S3 Classic | Garmin Fenix |
