# Supplementary material for: Predicted preference conjoint analysis
Source: PLoS One. 2021 Aug 26;16(8):e0256010. doi: 10.1371/journal.pone.0256010 (PMC8389521; doi:10.1371/journal.pone.0256010)
Supplement: S2 Questionnaire — (PDF) [file pone.0256010.s008.pdf]

Default Question Block

Welcome to our study!

We are academic researchers who are interested in how people make decisions about new products. In this study, we will ask you to choose between two comparable **streaming media sticks**.

Imagine that you are shopping for a streaming media stick. We will show you **20 pairs** of streaming media sticks, and for each pair we will ask you to choose the product you prefer more. Products will be described as **combinations of features**. Some products will be hypothetical and some will be real.

This study uses a specific method called **Bayesian Truth Serum** which is developed at MIT. Based on your answers, the Bayesian Truth Serum algorithm **computes a score** for every participant.

THE BETTER YOU PREDICT HOW OTHERS WILL CHOOSE, THE HIGHER YOUR SCORE WILL BE.

Participants whose scores are in **the top 50 percent** will receive an **additional reward of $1.00 on** top of the $1.50 for completing the questionnaire.

Informed consent:

By answering the following questions, you are participating in a research study being performed by scientists at the MIT Sloan School of Management. If you have questions about this research, please contact Sonja Radas at sradas@mit.edu. Your participation in this research is completely voluntary. You may decline to answer any or all of the following questions, and decline further

participation at any time, without adverse consequences. However, you will be paid only if the survey is completed. Your anonymity is assured; the researchers who have requested your participation will not receive any personal information about you.

Do you agree?

Yes, I am at least 18 years of age, have read and understand the explanation provided to me and voluntarily agree to participate in this study.

Do you already own a streaming media player?

Yes

No

Is that your first streaming media player?

Yes

No

Are you planning to buy a media player in near future?

Yes

No

Please rate the following statements according to how much you agree with them.

	Strongly Disagree	Disagree	Neither Agree nor Disagree	Agree	Strongly Agree
People around me ask me for advice in matters of technology	○	○	○	○	○
I am selective about what I watch	○	○	○	○	○
People around me ask me for advice about media choice	○	○	○	○	○

Do you tend to share information and discuss new technology and gadgets with friends, relatives, coworkers, etc.?

Yes

No

Approximately with how many people do you share information and discuss technology and new gadgets?

	0	3	6	9	12	15	18	21	24	27	30
Number of people with whom you discuss technology and new gadgets											

The product we are investigating in this study is a **streaming media stick.** This product can be plugged into any HDTV and connected to the existing WiFi network, thus enabling streaming of video and music content from online services. Streaming media sticks differ as to their price and capabilities.

Examples of existing streaming media sticks:

Image removed

The attributes that will be addressed in this study are **price, image resolution** (which can be 4 K at the high end, or 1080 p)**, popular content providers, ability to use the player in hotel rooms and dorms, and inclusion of remote control with the device.** Except for these attributes, the players that we will show you can be considered **basically the same on all other**

attributes.

Each of the media players we will show you supports the following list of favorite content providers: Netflix, Hulu, Crackle, Sling TV, PlayStation Vue, HBO, SHOWTIME, PBS, PBS Kids, WatchDisney, WatchESPN, FOX NOW, YouTube, Plex, Spotify, PlayOn and Pandora.

Other popular providers such as Amazon Video, Amazon Music, FandangoNOW (formerly M-GO), VUDU, Google Play, DIRECTV NOW **may or may not be supported**, depending on the product. None of the products we will show you supports iTunes.

Image removed

In the next section you will be presented with twenty pairs of different streaming media sticks, and for each pair we will ask you to choose the product you prefer.

Block 1

Please read the descriptions of two streaming media players. Players are presented as **combination of attributes**. Imagine that other that the differences shown in the table below, products are essentially the same.

We will ask you to choose the product you prefer more.

	**product A**	**product B**
Price	**$50**<br>**$$$$$**	**$40**<br>**$$$$**

Resolution	***4 K***	***4 K***
Remote	***Point of sight***	***Point anywhere***
Can be easily used in dorms and hotels	***YES***	***YES***
Extra channels	Amazon +DIRECTV	AMAZON+FVG

WHICH ONE OF THE TWO PRODUCTS DO YOU PREFER?

PRODUCT A

PRODUCT B

In your opinion, which percentage of other respondents will make the same choice as you?

	0	10	20	30	40	50	60	70	80	90	100
	0	10	20	30	40	50	60	70	80	90	100
Percentage of those who will make the same choice											

Block 2

Please read the descriptions of two streaming media players. Players are presented as **combination of attributes**. Imagine that other that the differences shown in the table below, products are essentially the same.

We will ask you to choose the product you prefer more.

	**product A**	**product B**
Price	**$40**<br>**$$$$**	**$50**<br>**$$$$$**
Resolution	***1080 p***	***4 K***
Remote	***Point of sight***	***NONE***<br>*(Other devices used instead such as mobile phone)*
Can be easily used in dorms and hotels	***NO***	

		YES
Extra channels	Amazon +DIRECTV+ FVG	FVG+DIRECTV

WHICH ONE OF THE TWO PRODUCTS DO YOU PREFER?

PRODUCT A

PRODUCT B

In your opinion, which percentage of other respondents will make the same choice as you?

	0	10	20	30	40	50	60	70	80	90	100
Percentage of those who will make the same choice											

Block 3

Please read the descriptions of two streaming media players. Players are presented as **combination of attributes**. Imagine that other that the differences shown in the table below, products are essentially the same.

We will ask you to choose the product you prefer more.

	product A	product B
Price	$50<br>$$$$$	$80<br>$$$$$$$$
Resolution	*4 K*	*4 K*
Remote	*Point anywhere*	*Point of sight*
Can be easily used in dorms and hotels	*YES*	*YES*
Extra channels	FVG+DIRECTV	Amazon +DIRECTV

WHICH ONE OF THE TWO PRODUCTS DO YOU PREFER?

PRODUCT A

PRODUCT B

In your opinion, which percentage of other respondents will make the same choice as you?

	0	10	20	30	40	50	60	70	80	90	100
Percentage of those who will make the same choice											

Block 4

Please read the descriptions of two streaming media players. Players are presented as **combination of attributes**. Imagine that other that the differences shown in the table below, products are essentially the same.

We will ask you to choose the product you prefer more.

	**product A**	**product B**
Price	**$70**<br>**$$$$$$$**	**$40**<br>**$$$$**
Resolution	***1080 p***	***4 K***
Remote	***NONE***<br>*(Other devices used instead*<br>*such as mobile phone)*	

		Point of sight
Can be easily used in dorms and hotels	***NO***	***YES***
Extra channels	AMAZON+FVG	FVG+DIRECTV

WHICH ONE OF THE TWO PRODUCTS DO YOU PREFER?

PRODUCT A

PRODUCT B

In your opinion, which percentage of other respondents will make the same choice as you?

	0	10	20	30	40	50	60	70	80	90	100
Percentage of those who will make the same choice											

Block 5

Please read the descriptions of two streaming media players. Players are presented as **combination of attributes.** Imagine that other that the differences shown in the table below, products are essentially the same.

We will ask you to choose the product you prefer more.

	product A	product B
Price	$80<br>$$$$$$$$	$50<br>$$$$$
Resolution	*1080 p*	*4 K*
Remote	*Point anywhere*	*Point of sight*
Can be easily used in dorms and hotels	*NO*	*YES*
Extra channels	Amazon +DIRECTV	AMAZON+FVG

WHICH ONE OF THE TWO PRODUCTS DO YOU PREFER?

PRODUCT A

PRODUCT B

In your opinion, which percentage of other respondents will make the same choice as you?

	0	10	20	30	40	50	60	70	80	90	100
Percentage of those who will make the same choice											

Block 6

Please read the descriptions of two streaming media players. Players are presented as **combination of attributes**. Imagine that other that the differences shown in the table below, products are essentially the same.

We will ask you to choose the product you prefer more.

	**product A**	**product B**
Price	**$40**<br>**$$$$**	**$50**<br>**$$$$$**
Resolution	***4 K***	***1080 p***
Remote		

	Point anywhere	***Point of sight***
Can be easily used in dorms and hotels	***YES***	***NO***
Extra channels	Amazon +DIRECTV	FVG+DIRECTV

WHICH ONE OF THE TWO PRODUCTS DO YOU PREFER?

PRODUCT A

PRODUCT B

In your opinion, which percentage of other respondents will make the same choice as you?

	0	10	20	30	40	50	60	70	80	90	100
Percentage of those who will make the same choice											

Block 7

Please read the descriptions of two streaming media players. Players are presented as **combination of attributes.** Imagine that other that the differences shown in the table below, products are essentially the same.

We will ask you to choose the product you prefer more.

	**product A**	**product B**
Price	**$40**<br>**$$$$**	**$50**<br>**$$$$$**
Resolution	***4 K***	***1080 p***
Remote	***Point of sight***	***Point anywhere***
Can be easily used in dorms and hotels	***NO***	***YES***
Extra channels	FVG+DIRECTV	Amazon +DIRECTV+ FVG

WHICH ONE OF THE TWO PRODUCTS DO YOU PREFER?

PRODUCT A

PRODUCT B

In your opinion, which percentage of other respondents will make the same choice as you?

	0	10	20	30	40	50	60	70	80	90	100
Percentage of those who will make the same choice											

Block 8

Please read the descriptions of two streaming media players. Players are presented as **combination of attributes**. Imagine that other that the differences shown in the table below, products are essentially the same.

We will ask you to choose the product you prefer more.

	**product A**	**product B**
Price	**$50**<br>**$$$$$**	**$80**<br>**$$$$$$$$**

Resolution	***1080 p***	***4 K***
Remote	***Point anywhere***	***NONE*** *(Other devices used instead such as mobile phone)*
Can be easily used in dorms and hotels	***YES***	***YES***
Extra channels	AMAZON+FVG	FVG+DIRECTV

WHICH ONE OF THE TWO PRODUCTS DO YOU PREFER?

PRODUCT A

PRODUCT B

In your opinion, which percentage of other respondents will make the same choice as you?

	0	10	20	30	40	50	60	70	80	90	100
Percentage of those who will make the same choice											

Block 9

Please read the descriptions of two streaming media players. Players are presented as **combination of attributes**. Imagine that other that the differences shown in the table below, products are essentially the same.

We will ask you to choose the product you prefer more.

	**product A**	**product B**
Price	**$50**<br>**$$$$$**	**$70**<br>**$$$$$$$**
Resolution	***4 K***	***1080 p***
Remote	***Point of sight***	***Point anywhere***
Can be easily used in dorms and hotels	***NO***	

		YES
Extra channels	AMAZON+FVG	FVG+DIRECTV

WHICH ONE OF THE TWO PRODUCTS DO YOU PREFER?

PRODUCT A

PRODUCT B

In your opinion, which percentage of other respondents will make the same choice as you?

	0	10	20	30	40	50	60	70	80	90	100
Percentage of those who will make the same choice											

Block 10

Please read the descriptions of two streaming media players. Players are presented as **combination of attributes**. Imagine that other that the differences shown in the table below, products are essentially the same.

We will ask you to choose the product you prefer more.

	**product A**	**product B**
Price	**$80**<br>**$$$$$$$$**	**$70**<br>**$$$$$$$**
Resolution	***1080 p***	***4 K***
Remote	***Point of sight***	***NONE***<br>*(Other devices used instead such as mobile phone)*
Can be easily used in dorms and hotels	***YES***	***NO***
Extra channels	FVG+DIRECTV	Amazon +DIRECTV

WHICH ONE OF THE TWO PRODUCTS DO YOU PREFER?

PRODUCT A

PRODUCT B

In your opinion, which percentage of other respondents will make the same choice as you?

	0	10	20	30	40	50	60	70	80	90	100
Percentage of those who will make the same choice											

Block 11

Please read the descriptions of two streaming media players. Players are presented as **combination of attributes**. Imagine that other that the differences shown in the table below, products are essentially the same.

We will ask you to choose the product you prefer more.

	**product A**	**product B**
Price	**$70**<br>**$$$$$$$**	**$50**<br>**$$$$$**
Resolution	***1080 p***	***1080 p***
Remote		

	Point of sight	***Point anywhere***
Can be easily used in dorms and hotels	***NO***	***NO***
Extra channels	AMAZON+FVG	Amazon +DIRECTV

WHICH ONE OF THE TWO PRODUCTS DO YOU PREFER?

PRODUCT A

PRODUCT B

In your opinion, which percentage of other respondents will make the same choice as you?

	0	10	20	30	40	50	60	70	80	90	100
Percentage of those who will make the same choice											

Block 12

Just to let you track your progress: you have seen 10 pairs of products.

Please read the descriptions of two streaming media players. Players are presented as **combination of attributes.** Imagine that other that the differences shown in the table below, products are essentially the same.

We will ask you to choose the product you prefer more.

	**product A**	**product B**
Price	**$80**<br>**$$$$$$$$**	**$70**<br>**$$$$$$$**
Resolution	***4 K***	***1080 p***
Remote	***Point anywhere***	***NONE***<br>*(Other devices used instead such as mobile phone)*
Can be easily used in dorms and hotels	***NO***	***YES***
Extra channels	FVG+DIRECTV	Amazon +DIRECTV

WHICH ONE OF THE TWO PRODUCTS DO YOU PREFER?

PRODUCT A

PRODUCT B

In your opinion, which percentage of other respondents will make the same choice as you?

	0	10	20	30	40	50	60	70	80	90	100
Percentage of those who will make the same choice											

Block 13

Please read the descriptions of two streaming media players. Players are presented as **combination of attributes**. Imagine that other that the differences shown in the table below, products are essentially the same.

We will ask you to choose the product you prefer more.

	**product A**	**product B**
Price	**$80**<br>**$$$$$$$$**	**$40**<br>**$$$$**

Resolution	***4 K***	***1080 p***
Remote	***Point anywhere***	***NONE***<br>*(Other devices used instead such as mobile phone)*
Can be easily used in dorms and hotels	***YES***	***NO***
Extra channels	AMAZON+FVG	Amazon +DIRECTV+ FVG

WHICH ONE OF THE TWO PRODUCTS DO YOU PREFER?

PRODUCT A

PRODUCT B

In your opinion, which percentage of other respondents will make the same choice as you?

	0	10	20	30	40	50	60	70	80	90	100
Percentage of those who will make the same choice											

Block 14

Please read the descriptions of two streaming media players. Players are presented as **combination of attributes**. Imagine that other that the differences shown in the table below, products are essentially the same.

We will ask you to choose the product you prefer more.

	**product A**	**product B**
Price	**$40**<br>**$$$$**	**$70**<br>**$$$$$$$**
Resolution	***1080 p***	***4 K***
Remote	***Point of sight***	***Point anywhere***

Can be easily used in dorms and hotels	***YES***	***NO***
Extra channels	Amazon +DIRECTV+ FVG	FVG+DIRECTV

WHICH ONE OF THE TWO PRODUCTS DO YOU PREFER?

PRODUCT A

PRODUCT B

In your opinion, which percentage of other respondents will make the same choice as you?

	0	10	20	30	40	50	60	70	80	90	100
Percentage of those who will make the same choice											

Block 15

Please read the descriptions of two streaming media players. Players are presented as **combination of attributes**. Imagine that other that the differences shown in the table below, products are essentially the same.

We will ask you to choose the product you prefer more.

	product A	product B
Price	**$40**<br>**$$$$**	**$70**<br>**$$$$$$$**
Resolution	***4 K***	***1080 p***
Remote	***NONE***<br>*(Other devices used instead such as mobile phone)*	***Point of sight***
Can be easily used in dorms and hotels	***NO***	***NO***
Extra channels	Amazon +DIRECTV+ FVG	Amazon +DIRECTV

WHICH ONE OF THE TWO PRODUCTS DO YOU PREFER?

PRODUCT A

PRODUCT B

In your opinion, which percentage of other respondents will make the same choice as you?

	0	10	20	30	40	50	60	70	80	90	100
Percentage of those who will make the same choice											

Block 16

Please read the descriptions of two streaming media players. Players are presented as **combination of attributes**. Imagine that other that the differences shown in the table below, products are essentially the same.

We will ask you to choose the product you prefer more.

	**product A**	**product B**
Price	**$40**<br>**$$$$**	**$70**<br>**$$$$$$$**
Resolution	***4 K***	***1080 p***
Remote		***NONE***<br>*(Other devices used*

	Point anywhere	*instead such as mobile phone)*
Can be easily used in dorms and hotels	***NO***	***YES***
Extra channels	Amazon +DIRECTV	FVG+DIRECTV

WHICH ONE OF THE TWO PRODUCTS DO YOU PREFER?

PRODUCT A

PRODUCT B

In your opinion, which percentage of other respondents will make the same choice as you?

	0	10	20	30	40	50	60	70	80	90	100
Percentage of those who will make the same choice											

Block 17

Please read the descriptions of two streaming media players. Players are presented as **combination of attributes.** Imagine that other that the differences shown in the table below, products are essentially the same.

We will ask you to choose the product you prefer more.

	**product A**	**product B**
Price	**$70**<br>**$$$$$$$**	**$40**<br>**$$$$**
Resolution	***4 K***	***1080 p***
Remote	***Point anywhere***	***Point of sight***
Can be easily used in dorms and hotels	***NO***	***YES***
Extra channels	Amazon<br>+DIRECTV+ FVG	Amazon<br>+DIRECTV

WHICH ONE OF THE TWO PRODUCTS DO YOU PREFER?

PRODUCT A

PRODUCT B

In your opinion, which percentage of other respondents will make the same choice as you?

	0	10	20	30	40	50	60	70	80	90	100
Percentage of those who will make the same choice											

Block 18

Please read the descriptions of two streaming media players. Players are presented as **combination of attributes**. Imagine that other that the differences shown in the table below, products are essentially the same.

We will ask you to choose the product you prefer more.

	**product A**	**product B**
Price	**$70**<br>**$$$$$$$**	**$80**<br>**$$$$$$$$**
Resolution		

	1080 p	***1080 p***
Remote	***Point of sight***	***NONE***<br>*(Other devices used instead such as mobile phone)*
Can be easily used in dorms and hotels	***YES***	***YES***
Extra channels	FVG+DIRECTV	AMAZON+FVG

WHICH ONE OF THE TWO PRODUCTS DO YOU PREFER?

PRODUCT A

PRODUCT B

In your opinion, which percentage of other respondents will make the same choice as you?

0 10 20 30 40 50 60 70 80 90 100

Percentage of those who will make the same choice

Block 6 again

Please read the descriptions of two streaming media players. Players are presented as **combination of attributes**. Imagine that other that the differences shown in the table below, products are essentially the same.

We will ask you to choose the product you prefer more.

	**product A**	**product B**
Price	**$40**<br>**$$$$**	**$50**<br>**$$$$$**
Resolution	***4 K***	***1080 p***
Remote	***Point anywhere***	***Point of sight***
Can be easily used in dorms and hotels		***NO***

	YES	
Extra channels	Amazon +DIRECTV	FVG+DIRECTV

WHICH ONE OF THE TWO PRODUCTS DO YOU PREFER?

PRODUCT A

PRODUCT B

In your opinion, which percentage of other respondents will make the same choice as you?

	0	10	20	30	40	50	60	70	80	90	100
Percentage of those who will make the same choice											

Block 19

Please read the descriptions of two streaming media players. Players are presented as **combination of attributes**. Imagine that other that the differences shown in the table below, products are essentially the same.

We will ask you to choose the product you prefer more.

	**product A**	**product B**
Price	**$70**<br>**$$$$$$$**	**$80**<br>**$$$$$$$$**
Resolution	***1080 p***	***1080 p***
Remote	***Point of sight***	***NONE***<br>*(Other devices used instead such as mobile phone)*
Can be easily used in dorms and hotels	***YES***	***YES***
Extra channels	FVG+DIRECTV	AMAZON+FVG

WHICH ONE OF THE TWO PRODUCTS DO YOU PREFER?

PRODUCT A

PRODUCT B

In your opinion, which percentage of other respondents will make the same choice as you?

	0	10	20	30	40	50	60	70	80	90	100
Percentage of those who will make the same choice											

Block 20

Please consider the three real streaming media sticks below. Each of the products supports the following list of favorite content providers: Netflix, Hulu, Crackle, Sling TV, PlayStation Vue, HBO, SHOWTIME, PBS, PBS Kids, WatchDisney, WatchESPN, FOX NOW, YouTube, Plex, Spotify, PlayOn and Pandora. They differ in price, resolution, remote control, ability to be used in hotels and dorms, and extra channel providers.

Imagine that you are shopping for a streaming media stick. Which product would be your **first choice**?

PLEASE INDICATE YOUR FIRST CHOICE

	ROKU	Chromecast ultra	ROKU Premiere
Price	$49.99 $$$$$	$69.99 $$$$$$$	$79.99 $$$$$$$$
Resolution	1080 p	4 K	4 K
Remote	Point anywhere	NONE	Point of sight
Can be easily used in dorms and hotels	YES	NO	YES
Extra channels	AMAZON+ FVG	AMAZON+ FVG	FVG+ DIRECTV

PRODUCT A -ROKU

PRODUCT B - Chromecast Ultra

PRODUCT C - ROKU Premiere

Which of the two remaing products is your second choice?

PRODUCT A - ROKU

PRODUCT B -Chromecast Ultra

PRODUCT C - ROKU Premiere

Block 21

Could you please indicate your gender?

Male

Female

Could you please indicate your age?

Under 18

18-24

25-44

45-64

Over 65

Could you please indicate your highest education level?

Elementary school

High school

Undergraduate college or university

Graduate school

PhD

How much overnight travel do you do?

More than 2 per week

Between 1 and 2 per week

Between 1 to 3 per month

Between 2 and 10 per year

Less than 2 per year

Could you please indicate your average monthly income

< 2,000

2,001 - 4,000

4,001 - 8,000

8,001 - 12,000

> 12,001

We'd love any comments you have about the experiment.

Powered by Qualtrics
