# Supplementary material for: Predicted preference conjoint analysis
Source: PLoS One. 2021 Aug 26;16(8):e0256010. doi: 10.1371/journal.pone.0256010 (PMC8389521; doi:10.1371/journal.pone.0256010)
Supplement: S3 Questionnaire — (PDF) [file pone.0256010.s009.pdf]

Default Question Block

Welcome to our study!

We are academic researchers who are interested in how people make decisions about new products.

We will show you 20 pairs of activity/fitness trackers, and for each pair we will ask you to choose the product you prefer more. Products will be described as combinations of features. Some product will be hypothetical and some will be real.

We will also ask you about your experience with activity trackers, and at the end we will ask you some basic demographic questions.

This study uses a specific method called **Bayesian Truth Serum** which is developed at **Massachusetts Institute of Technology.**

- Based on your answers, the Bayesian Truth Serum algorithm **computes a score** for every participant.
- Score is **higher** for people who **answer truthfully** and who are **good at predicting** what others will choose.
- Participants whose scores are in the **top 25 percent** will receive an **additional reward of $1.00** on top of the $1.50 for completing the questionnaire.

Please do not take this survey more than once.

Informed consent:

By answering the following questions, you are participating in a research study being performed by scientists at the MIT Sloan School of Management. If you have questions about this research,

please contact Sonja Radas at sradas@mit.edu. Your participation in this research is completely voluntary. You may decline to answer any or all of the following questions, and decline further participation at any time, without adverse consequences. However, you will be paid only if the survey is completed. Your anonymity is assured; the researchers who have requested your participation will not receive any personal information about you.

Do you agree?

Yes, I am at least 18 years of age, have read and understand the explanation provided to me and voluntarily agree to participate in this study.

Do you already own a fitness/activity tracking device?

Yes

No

Is that your first fitness tracker?

Yes

No

Are you planning to buy a fitness tracker in near future?

Yes

No

Please rate the following statements according to how much you agree with them.

	Strongly Disagree	Disagree	Neither Agree nor Disagree	Agree	Strongly Agree
People around me ask me for advice in matters of technology	○	○	○	○	○
I follow the market for fitness trackers	○	○	○	○	○
I like discussing technology with people around me	○	○	○	○	○

	Strongly Disagree	Disagree	Neither Agree nor Disagree	Agree	Strongly Agree
Fitness is an important part of my life	○	○	○	○	○

In an average week, how many hours do you work out?

Less than 2 hours

Between 2 and 3 hours

Between 3 and 4 hours

Between 4 and 5 hours

More than 5 hours

On average, how many steps do you make daily?

Below 3000

Between 3000 and 5000

Between 5000 and 8000

Between 8000 and 10000

Over 10000

Approximately with how many people do you share information and discuss technology and new gadgets?

	0	3	6	9	12	15	18	21	24	27	30
Number of people with whom you discuss technology and new gadgets											

Imagine that you are shopping for a fitness tracker.

We will show you 20 pairs of possible products that will be presented as combinations of features, and for each pair we will ask you to choose the product

that you prefer. The fitness trackers we will show you are bracelets made of flexible elastomer material, which can come in several different colors. All trackers belong to the same brand.

Each of the products that we will show you automatically tracks steps, calories burned and distance travelled. Each one syncs wirelessly with your mobile phone and/or computer, where it uses the free app to display the stats and activity data. All the products that we will show you have sleep tracking and silent alarm, and rechargeable battery.

Here are some examples of existing bracelet fitness trackers:

Image removed

The trackers that we will show you can be considered basically the same except that they differ in following attributes: ability to **track heart rate** during your day as well as in workouts, **workout tracking** with real-time stats, ability to **connect with GPS** device in you phone, **water resistance**, **display and notifications** (some devices have OLED display for text and call notifications, while some have only small LED lights which flash when text or call was received), **battery life**, and **price**.

In the next section you will be presented with twenty pairs of different fitness trackers, and for each pair we will ask you to choose the product you prefer.

Block 1

Please read the descriptions of two fitness trackers. Trackers are presented as **combination of attributes**. Imagine that other that the differences shown in the table below, products are essentially the same.

We will ask you to choose the product you prefer more.

	product A	product B
Heart rate tracking during day and workouts	YES	NO
Connect to your phone's GPS	NO	YES
Track workouts with real time stats	NO	YES
Water resistance	Splash and rain resistant	Swim-proof
Display and notifications	OLED display shows **call** and **text** notifications, and **calendar** alerts	OLED display shows **call** and **text** notifications
Battery life	14 days	7 days
Price	$99	$129

WHICH ONE OF THE TWO PRODUCTS DO YOU PREFER?

PRODUCT A

PRODUCT B

In your opinion, which percentage of other respondents will make the same choice as you?

	0	10	20	30	40	50	60	70	80	90	100
Percentage of those who will make the same choice											

Block 2

Please read the descriptions of two fitness trackers. Trackers are presented as **combination of attributes**. Imagine that other that the differences shown in the table below, products are essentially the same.

We will ask you to choose the product you prefer more.

	product A	product B
Heart rate tracking during day and workouts	NO	YES
Connect to your phone's GPS	YES	NO
Track workouts with real time stats	NO	YES
Water resistance	Swim-proof	Splash and rain resistant
Display and notifications	OLED display shows **call** and **text** notifications, and **calendar** alerts OLED display shows **call** and **text** notifications, and **calendar** alerts	OLED display shows **call** and **text** notifications
Battery life	5 days	7 days
Price	$99	$129

WHICH ONE OF THE TWO PRODUCTS DO YOU PREFER?

PRODUCT A PRODUCT B

In your opinion, which percentage of other respondents will make the same choice as you?

0 10 20 30 40 50 60 70 80 90 100

	0	10	20	30	40	50	60	70	80	90	100
Percentage of those who will make the same choice											

Block 3

Please read the descriptions of two fitness trackers. Trackers are presented as **combination of attributes**. Imagine that other that the differences shown in the table below, products are essentially the same.

We will ask you to choose the product you prefer more.

	product A	product B
Heart rate tracking during day and workouts	NO	YES
Connect to your phone's GPS	NO	YES
Track workouts with real time stats	NO	YES
Water resistance	Swim-proof	Splash and rain resistant
Display and notifications	OLED display shows **call** and **text** notifications, and **calendar** alerts	No display, **vibration** and **small LED lights** alert you to check your phone for new calls and text
Battery life	**5** days	**7** days
Price	**$129**	**$99**

WHICH ONE OF THE TWO PRODUCTS DO YOU PREFER?

PRODUCT A PRODUCT B

In your opinion, which percentage of other respondents will make the same choice as you?

0 10 20 30 40 50 60 70 80 90 100

	0	10	20	30	40	50	60	70	80	90	100
Percentage of those who will make the same choice											

Block 4

Please read the descriptions of two fitness trackers. Trackers are presented as **combination of attributes**. Imagine that other that the differences shown in the table below, products are essentially the same.

We will ask you to choose the product you prefer more.

	product A	product B
Heart rate tracking during day and workouts	YES	YES
Connect to your phone's GPS	NO	YES
Track workouts with real time stats	NO	YES
Water resistance	Swim-proof	Swim-proof
Display and notifications	OLED display shows **call** and **text** notifications,	OLED display shows **call** and **text** notifications, and **calendar** alerts
Battery life	5 days	14 days
Price	$79	$99

WHICH ONE OF THE TWO PRODUCTS DO YOU PREFER?

PRODUCT A PRODUCT B

In your opinion, which percentage of other respondents will make the same choice as you?

0 10 20 30 40 50 60 70 80 90 100

	0	10	20	30	40	50	60	70	80	90	100
Percentage of those who will make the same choice											

Block 5

Please read the descriptions of two fitness trackers. Trackers are presented as **combination of attributes**. Imagine that other that the differences shown in the table below, products are essentially the same.

We will ask you to choose the product you prefer more.

	product A	product B
Heart rate tracking during day and workouts	NO	YES
Connect to your phone's GPS	NO	YES
Track workouts with real time stats	YES	NO
Water resistance	Swim-proof	Splash and rain resistant
Display and notifications	OLED display shows **call** and **text** notifications, and **calendar** alerts	OLED display shows **call** and **text** notifications,
Battery life	**7** days	**14** days
Price	**$79**	**$129**

WHICH ONE OF THE TWO PRODUCTS DO YOU PREFER?

PRODUCT A PRODUCT B

In your opinion, which percentage of other respondents will make the same choice as you?

0 10 20 30 40 50 60 70 80 90 100

	0	10	20	30	40	50	60	70	80	90	100
Percentage of those who will make the same choice											

Block 6

Please read the descriptions of two fitness trackers. Trackers are presented as **combination of attributes**. Imagine that other that the differences shown in the table below, products are essentially the same.

We will ask you to choose the product you prefer more.

	product A	product B
Heart rate tracking during day and workouts	YES	NO
Connect to your phone's GPS	NO	YES
Track workouts with real time stats	NO	YES
Water resistance	Swim-proof	Splash and rain resistant
Display and notifications	No display, **vibration** and **small LED lights** alert you to check your phone for new calls and text	OLED display shows **call** and **text** notifications,
Battery life	7 days	5 days
Price	$99	$79

WHICH ONE OF THE TWO PRODUCTS DO YOU PREFER?

PRODUCT A PRODUCT B

In your opinion, which percentage of other respondents will make the same choice as you?

	0	10	20	30	40	50	60	70	80	90	100
Percentage of those who will make the same choice											

Block 7

Please read the descriptions of two fitness trackers. Trackers are presented as **combination of attributes**. Imagine that other that the differences shown in the table below, products are essentially the same.

We will ask you to choose the product you prefer more.

	**product A**	**product B**
**Heart rate tracking during day and workouts**	YES	NO
**Connect to your phone's GPS**	YES	NO
**Track workouts with real time stats**	NO	YES
**Water resistance**	Swim-proof	Splash and rain resistant
**Display and notifications**	OLED display shows **call** and **text** notifications,	No display, **vibration** and **small LED lights** alert you to check your phone for new calls and text
**Battery life**	**7** days	**14** days
**Price**	**$129**	**$99**

WHICH ONE OF THE TWO PRODUCTS DO YOU PREFER?

PRODUCT A PRODUCT B

In your opinion, which percentage of other respondents will make the same choice as you?

	0	10	20	30	40	50	60	70	80	90	100
Percentage of those who will make the same choice											

Block 8

Please read the descriptions of two fitness trackers. Trackers are presented as **combination of attributes**. Imagine that other that the differences shown in the table below, products are essentially the same.

We will ask you to choose the product you prefer more.

	**product A**	**product B**
**Heart rate tracking during day and workouts**	NO	YES
**Connect to your phone's GPS**	YES	NO
**Track workouts with real time stats**	NO	YES
	Splash and rain resistant	Swim-proof
**Display and notifications**	OLED display shows **call** and **text** notifications, and **calendar** alerts	OLED display shows **call** and **text** notifications,
**Battery life**	**7** days	**5**
**Price**	**$79**	**$129**

WHICH ONE OF THE TWO PRODUCTS DO YOU PREFER?

PRODUCT A PRODUCT B

In your opinion, which percentage of other respondents will make the same choice as you?

0 10 20 30 40 50 60 70 80 90 100

	0	10	20	30	40	50	60	70	80	90	100
Percentage of those who will make the same choice											

Block 9

Please read the descriptions of two fitness trackers. Trackers are presented as **combination of attributes**. Imagine that other that the differences shown in the table below, products are essentially the same.

We will ask you to choose the product you prefer more.

	product A	product B
Heart rate tracking during day and workouts	YES	NO
Connect to your phone's GPS	NO	YES
Track workouts with real time stats	NO	YES
Water resistance	Splash and rain resistant	Swim-proof
Display and notifications	OLED display shows **call** and **text** notifications, and **calendar** alerts	OLED display shows **call** and **text** notifications,
Battery life	**7** days	**14** days
Price	**$129**	**$99**

WHICH ONE OF THE TWO PRODUCTS DO YOU PREFER?

PRODUCT A PRODUCT B

In your opinion, which percentage of other respondents will make the same choice as you?

0 10 20 30 40 50 60 70 80 90 100

	0	10	20	30	40	50	60	70	80	90	100
Percentage of those who will make the same choice											

Block 10

Please read the descriptions of two fitness trackers. Trackers are presented as **combination of attributes**. Imagine that other that the differences shown in the table below, products are essentially the same.

We will ask you to choose the product you prefer more.

	product A	product B
Heart rate tracking during day and workouts	YES	NO
Connect to your phone's GPS	NO	YES
Track workouts with real time stats	YES	NO
Water resistance	Swim-proof	Splash and rain resistant
Display and notifications	OLED display shows **call** and **text** notifications,	No display, **vibration** and **small LED lights** alert you to check your phone for new calls and text
Battery life	**7** days	**5** days
Price	**$99**	**$129**

WHICH ONE OF THE TWO PRODUCTS DO YOU PREFER?

PRODUCT A PRODUCT B

In your opinion, which percentage of other respondents will make the same choice as you?

	0	10	20	30	40	50	60	70	80	90	100
Percentage of those who will make the same choice											

Block 11

Please read the descriptions of two fitness trackers. Trackers are presented as **combination of attributes**. Imagine that other that the differences shown in the table below, products are essentially the same.

We will ask you to choose the product you prefer more.

	product A	product B
Heart rate tracking during day and workouts	NO	YES
Connect to your phone's GPS	NO	YES
Track workouts with real time stats	NO	YES
Water resistance	Swim-proof	Splash and rain resistant
Display and notifications	OLED display shows **call** and **text** notifications,	No display, **vibration** and **small LED lights** alert you to check your phone for new calls and text
Battery life	14 days	7 days
Price	$99	$129

WHICH ONE OF THE TWO PRODUCTS DO YOU PREFER?

PRODUCT A PRODUCT B

In your opinion, which percentage of other respondents will make the same choice as you?

	0	10	20	30	40	50	60	70	80	90	100
Percentage of those who will make the same choice											

Block 12

Please read the descriptions of two fitness trackers. Trackers are presented as **combination of attributes**. Imagine that other that the differences shown in the table below, products are essentially the same.

We will ask you to choose the product you prefer more.

	product A	product B
Heart rate tracking during day and workouts	NO	YES
Connect to your phone's GPS	NO	YES
Track workouts with real time stats	YES	NO
Water resistance	Swim-proof	Splash and rain resistant
Display and notifications	OLED display shows **call** and **text** notifications, and **calendar** alerts	No display, **vibration** and **small LED lights** alert you to check your phone for new calls and text
Battery life	**5** days	**5** days
Price	**$79**	**$79**

WHICH ONE OF THE TWO PRODUCTS DO YOU PREFER?

PRODUCT A PRODUCT B

In your opinion, which percentage of other respondents will make the same choice as you?

0 10 20 30 40 50 60 70 80 90 100

	0	10	20	30	40	50	60	70	80	90	100
Percentage of those who will make the same choice											

Block 13

Please read the descriptions of two fitness trackers. Trackers are presented as **combination of attributes**. Imagine that other that the differences shown in the table below, products are essentially the same.

We will ask you to choose the product you prefer more.

	product A	product B
Heart rate tracking during day and workouts	YES	NO
Connect to your phone's GPS	YES	NO
Track workouts with real time stats	YES	NO
Water resistance	Splash and rain resistant	Swim-proof
Display and notifications	OLED display shows **call** and **text** notifications,	No display, **vibration** and **small LED lights** alert you to check your phone for new calls and text
Battery life	**5** days	**7** days
Price	**$99**	**$99**

WHICH ONE OF THE TWO PRODUCTS DO YOU PREFER?

PRODUCT A PRODUCT B

In your opinion, which percentage of other respondents will make the same choice as you?

	0	10	20	30	40	50	60	70	80	90	100
Percentage of those who will make the same choice											

Block 14

Please read the descriptions of two fitness trackers. Trackers are presented as **combination of attributes**. Imagine that other that the differences shown in the table below, products are essentially the same.

We will ask you to choose the product you prefer more.

	**product A**	**product B**
**Heart rate tracking during day and workouts**	NO	YES
**Connect to your phone's GPS**	YES	NO
**Track workouts with real time stats**	NO	YES
**Water resistance**	Swim-proof	Swim-proof
**Display and notifications**	OLED display shows **call** and **text** notifications,	No display, **vibration** and **small LED lights** alert you to check your phone for new calls and text
**Battery life**	7 days	5 days
**Price**	**$99**	**$79**

WHICH ONE OF THE TWO PRODUCTS DO YOU PREFER?

PRODUCT A PRODUCT B

In your opinion, which percentage of other respondents will make the same choice as you?

	0	10	20	30	40	50	60	70	80	90	100
Percentage of those who will make the same choice											

Block 15

Please read the descriptions of two fitness trackers. Trackers are presented as **combination of attributes**. Imagine that other that the differences shown in the table below, products are essentially the same.

We will ask you to choose the product you prefer more.

	product A	product B
**Heart rate tracking during day and workouts**	NO	YES
**Connect to your phone's GPS**	NO	YES
**Track workouts with real time stats**	YES	NO
**Water resistance**	Swim-proof	Splash and rain resistant
**Display and notifications**	OLED display shows **call** and **text** notifications, and **calendar** alerts	No display, **vibration** and **small LED lights** alert you to check your phone for new calls and text
**Battery life**	5 days	5 days
**Price**	**$79**	**$79**

WHICH ONE OF THE TWO PRODUCTS DO YOU PREFER?

PRODUCT A PRODUCT B

In your opinion, which percentage of other respondents will make the same choice as you?

0 10 20 30 40 50 60 70 80 90 100

	0	10	20	30	40	50	60	70	80	90	100
Percentage of those who will make the same choice											

Block 16

Please read the descriptions of two fitness trackers. Trackers are presented as **combination of attributes**. Imagine that other that the differences shown in the table below, products are essentially the same.

We will ask you to choose the product you prefer more.

	product A	product B
Heart rate tracking during day and workouts	YES	YES
Connect to your phone's GPS	YES	YES
Track workouts with real time stats	NO	NO
Water resistance	Splash and rain resistant	Splash and rain resistant
Display and notifications	OLED display shows **call** and **text** notifications,	No display, **vibration** and **small LED lights** alert you to check your phone for new calls and text
Battery life	7 days	5 days
Price	**$99**	**$79**

WHICH ONE OF THE TWO PRODUCTS DO YOU PREFER?

PRODUCT A PRODUCT B

In your opinion, which percentage of other respondents will make the same choice as you?

	0	10	20	30	40	50	60	70	80	90	100
Percentage of those who will make the same choice											

Block 17

Please read the descriptions of two fitness trackers. Trackers are presented as **combination of attributes**. Imagine that other that the differences shown in the table below, products are essentially the same.

We will ask you to choose the product you prefer more.

	product A	product B
Heart rate tracking during day and workouts	NO	YES
Connect to your phone's GPS	NO	YES
Track workouts with real time stats	YES	NO
Water resistance	Splash and rain resistant	Swim-proof
Display and notifications	OLED display shows **call** and **text** notifications,	No display, **vibration** and **small LED lights** alert you to check your phone for new calls and text
Battery life	14 days	7 days
Price	$129	$79

WHICH ONE OF THE TWO PRODUCTS DO YOU PREFER?

PRODUCT A PRODUCT B

In your opinion, which percentage of other respondents will make the same choice as you?

	0	10	20	30	40	50	60	70	80	90	100
Percentage of those who will make the same choice											

Block 18

Please read the descriptions of two fitness trackers. Trackers are presented as **combination of attributes**. Imagine that other that the differences shown in the table below, products are essentially the same.

We will ask you to choose the product you prefer more.

	product A	product B
Heart rate tracking during day and workouts	YES	YES
Connect to your phone's GPS	NO	NO
Track workouts with real time stats	YES	NO
Water resistance	Splash and rain resistant	Splash and rain resistant
Display and notifications	OLED display shows **call** and **text** notifications,	No display, **vibration** and **small LED lights** alert you to check your phone for new calls and text
Battery life	5 days	5 days
Price	**$99**	**$79**

WHICH ONE OF THE TWO PRODUCTS DO YOU PREFER?

PRODUCT A PRODUCT B

In your opinion, which percentage of other respondents will make the same choice as you?

	0	10	20	30	40	50	60	70	80	90	100
Percentage of those who will make the same choice											

Block 6 again

Please read the descriptions of two fitness trackers. Trackers are presented as **combination of attributes**. Imagine that other that the differences shown in the table below, products are essentially the same.

We will ask you to choose the product you prefer more.

	product A	product B
Heart rate tracking during day and workouts	YES	NO
Connect to your phone's GPS	NO	YES
Track workouts with real time stats	NO	YES
Water resistance	Swim-proof	Splash and rain resistant
Display and notifications	No display, **vibration** and **small LED lights** alert you to check your phone for new calls and text	OLED display shows **call** and **text** notifications,
Battery life	7 days	5 days
Price	$99	$79

WHICH ONE OF THE TWO PRODUCTS DO YOU PREFER?

PRODUCT A

PRODUCT B

In your opinion, which percentage of other respondents will make the same choice as you?

	0	10	20	30	40	50	60	70	80	90	100
Percentage of those who will make the same choice											

Block 19

Please read the descriptions of two fitness trackers. Trackers are presented as **combination of attributes**. Imagine that other that the differences shown in the table below, products are essentially the same.

We will ask you to choose the product you prefer more.

	product A	product B
Heart rate tracking during day and workouts	YES	YES
Connect to your phone's GPS	NO	NO
Track workouts with real time stats	YES	NO
Water resistance	Splash and rain resistant	Splash and rain resistant
Display and notifications	OLED display shows **call** and **text** notifications,	No display, **vibration** and **small LED lights** alert you to check your phone for new calls and text
Battery life	5 days	5 days
Price	$99	$79

WHICH ONE OF THE TWO PRODUCTS DO YOU PREFER?

PRODUCT A PRODUCT B

In your opinion, which percentage of other respondents will make the same choice as you?

	0	10	20	30	40	50	60	70	80	90	100
Percentage of those who will make the same choice											

Block 20

Here we show you three real fitness trackers. Each of the products automatically tracks steps, calories burned and distance travelled. Each one syncs wirelessly with your mobile phone and/or computer, where it uses the free app to display the stats and activity data. All the products have sleep tracking and silent alarm, and rechargeable battery.

Imagine that you are shopping for a fitness tracker. Which product would be your **first choice**?

	Fotbit Alta	Fitbit Flex	Fitbit Charge
Heart rate tracking during day and workouts	NO	NO	YES
Connect to your phone's GPS	NO	NO	YES
Track workouts with real time stats	NO	NO	YES
Water resistance	Splash and rain resistant	Swim-proof	Splash and rain resistant
Display and notifications	OLED display shows **call** and **text** notifications, and **calendar** alerts	No display, **vibration** and **small LED lights** alert you to check your phone for new calls and text	OLED display shows **call** and **text** notifications, and **calendar** alerts
Battery life	5 days	5 days	5 days
Price	**$99**	**$79**	**$129**

PLEASE INDICATE YOUR FIRST CHOICE

PRODUCT A

PRODUCT B

PRODUCT C

WHICH OF THE TWO REMAINING PRODUCTS WOULD BE YOUR SECOND CHOICE?

PRODUCT A	PRODUCT B	PRODUCT C

Block 21

Could you please indicate your gender?

Male

Female

Could you please indicate your age?

Under 18

18-24

25-44

45-64

Over 65

Could you please indicate your highest education level?

Elementary school

High school

Undergraduate college or university

Graduate school

PhD

How much overnight travel do you do?

More than 2 per week

Between 1 and 2 per week

Between 1 to 3 per month

Between 2 and 10 per year

Less than 2 per year

Could you please indicate your average monthly household income

< 2,000

2,001 - 4,000

4,001 - 8,000

8,001 - 12,000

> 12,001

We'd love any comments you have about the experiment.

Powered by Qualtrics
