# Supplementary material for: Predicted preference conjoint analysis
Source: PLoS One. 2021 Aug 26;16(8):e0256010. doi: 10.1371/journal.pone.0256010 (PMC8389521; doi:10.1371/journal.pone.0256010)
Supplement: S5 Questionnaire — (PDF) [file pone.0256010.s011.pdf]

Default Question Block

Welcome to our study!

We will show you 8 sets of **smartwatches** and ask you to choose your favorite.
We are also interested in how much you know about other people, so we will ask you to **predict what others will choose**.

You will be paid **$0.25 to complete the task**, but you can earn more:

- If you are a **good predictor,**
- If you give us **truthful answers**.

This study uses a specific method called Bayesian Truth Serum which is developed at Massachusetts Institute of Technology (MIT). Based on your answers, the Bayesian Truth Serum algorithm computes a score for every participant. Participants whose scores are in **the top 50 percent** will receive a bonus **of $0.25.**

Informed consent:
This HIT is part of a MIT scientific research project. Your decision to complete this HIT is voluntary. There is no way for us to identify you. The only information we will have, in addition to your responses, is the time at which you completed the survey. The results of the research may be presented at scientific meetings or published in scientific journals. Clicking on the 'SUBMIT' button on the bottom of this page indicates that you are at least 18 years of age and agree to complete this HIT voluntarily. If you have any questions about this study, please contact sradas@mit.edu

SUBMIT (I am at least 18 years of age, have read and understand the explanation provided to me and voluntarily agree to participate in this study).

Do you already own a smartwatch?

Yes

No

Is that your first smartwatch?

Yes

No

Are you planning to buy a smartwatch in near future?

Yes

No

We will show you 8 sets of smartwatches. To make your task easier, we will highlight the differences among the products by emphasizing several features that most people find important.

In each set we will ask you to choose your preferred product, and to answer questions about the choices and predictions of other people.

Block real1

Which smartwatch of the three shown below would you choose?

	Apple 2	Apple 3	Samsung Gear S3 Classic
**Fitness tracking**	Yes	Yes	No, does not have it
**Health/heart tracking**	Basic (heart rate, pulse)	Standard (heart rate, pulse, BPM)	Basic (heart rate, pulse)
**Price**	$200	$300	$200

PLEASE INDICATE YOUR CHOICE

PRODUCT A PRODUCT B PRODUCT C

What percentage of other respondents will choose products A and B?

	0	10	20	30	40	50	60	70	80	90	100
% who will choose Product A											
% who will choose Product B											

Block real2

Which smartwatch of the three shown below would you choose?

	Apple 4	Samsung Galaxy Watch	Garmin Fenix 5
**Fitness tracking**	Yes	Yes	Yes, advanced
**Health/heart tracking**	Advanced (heart rate, pulse, BPM, ECG, irregular heart rate alert)	Standard (heart rate, pulse, BPM)	Standard (heart rate, pulse, BPM)
**Price**	$400	$300	$400

PLEASE INDICATE YOUR CHOICE

PRODUCT A PRODUCT B PRODUCT C

What percentage of other respondents will choose products A and B?

	0	10	20	30	40	50	60	70	80	90	100
% who will choose Product A											
% who will choose Product B											

Block real3

Which smartwatch of the three shown below would you choose?

	Apple 3	Apple 4	Samsung Galaxy Watch
**Fitness tracking**	Yes	Yes	Yes
**Health/heart tracking**	Standard (heart rate, pulse, BPM)	Advanced (heart rate, pulse, BPM, ECG, irregular heart rate alert)	Standard (heart rate, pulse, BPM)
**Price**	$300	$400	$300

PLEASE INDICATE YOUR CHOICE

PRODUCT A PRODUCT B PRODUCT C

What percentage of other respondents will choose products A and B?

	0	10	20	30	40	50	60	70	80	90	100
% who will choose Product A											
% who will choose Product B											

Block real4

Which smartwatch of the three shown below would you choose?

	Samsung Galaxy Watch	Samsung Gear S3 Classic	Garmin Fenix 5
**Fitness tracking**	Yes	No, does not have it	Yes, advanced
**Health/heart tracking**	Standard (heart rate, pulse, BPM)	Basic (heart rate, pulse)	Standard (heart rate, pulse, BPM)
**Price**	$300	$200	$400

PLEASE INDICATE YOUR CHOICE

PRODUCT A PRODUCT B PRODUCT C

What percentage of other respondents will choose products A and B?

	0	10	20	30	40	50	60	70	80	90	100
% who will choose Product A											
% who will choose Product B											

Block real5

Which smartwatch of the three shown below would you choose?

	Apple 3	Samsung Galaxy Watch	Samsung Gear S3 Classic
**Fitness tracking**	Yes	Yes	No, does not have it
**Health/heart tracking**	Standard (heart rate, pulse, BPM)	Standard (heart rate, pulse, BPM)	Basic (heart rate, pulse)
**Price**	$300	$300	$200

PLEASE INDICATE YOUR CHOICE

PRODUCT A PRODUCT B PRODUCT C

What percentage of other respondents will choose products A and B?

	0	10	20	30	40	50	60	70	80	90	100
% who will choose Product A											
% who will choose Product B											

Block real6

Which smartwatch of the three shown below would you choose?

	Apple 3	Samsung Gear S3 Classic	Garmin Fenix 5
**Fitness tracking**	Yes	No, does not have it	Yes, advanced
**Health/heart tracking**	Standard (heart rate, pulse, BPM)	Basic (heart rate, pulse)	Standard (heart rate, pulse, BPM)
**Price**	$300	$200	$400

PLEASE INDICATE YOUR CHOICE

PRODUCT A PRODUCT B PRODUCT C

What percentage of other respondents will choose products A and B?

	0	10	20	30	40	50	60	70	80	90	100
% who will choose Product A											
% who will choose Product B											

Block real7

Which smartwatch of the three shown below would you choose?

	Apple 3	Apple 4	Garmin Fenix 5
**Fitness tracking**	Yes	Yes	Yes, advanced
**Health/heart tracking**	Standard (heart rate, pulse, BPM)	Advanced (heart rate, pulse, BPM, ECG, irregular heart rate alert)	Standard (heart rate, pulse, BPM)
**Price**	$300	$400	$400

PLEASE INDICATE YOUR CHOICE

PRODUCT A PRODUCT B PRODUCT C

What percentage of other respondents will choose products A and B?

	0	10	20	30	40	50	60	70	80	90	100
% who will choose Product A											
% who will choose Product B											

Block real8

Which smartwatch of the three shown below would you choose?

	Apple 4	Samsung Gear S3 Classic	Garmin Fenix 5
**Fitness tracking**	Yes	No, does not have it	Yes, advanced
**Health/heart tracking**	Advanced (heart rate, pulse, BPM, ECG, irregular heart rate alert)	Basic (heart rate, pulse)	Standard (heart rate, pulse, BPM)
**Price**	$400	$200	$400

PLEASE INDICATE YOUR CHOICE

PRODUCT A PRODUCT B PRODUCT C

What percentage of other respondents will choose products A and B?

	0	10	20	30	40	50	60	70	80	90	100
Product A											
Product B											

Block demographic

Could you please indicate your gender?

Male

Female

Could you please indicate your age?

Under 18

18-24

25-44

45-64

Over 65

Could you please indicate your highest education level?

Elementary school

High school

Undergraduate college or university

Graduate school

PhD

We'd love any comments you have about the experiment.

Powered by Qualtrics
