# Supplementary material for: Predicted preference conjoint analysis
Source: PLoS One. 2021 Aug 26;16(8):e0256010. doi: 10.1371/journal.pone.0256010 (PMC8389521; doi:10.1371/journal.pone.0256010)
Supplement: S1 Code — (DOCX) [file pone.0256010.s012.docx]

**Hierarchical Bayesian estimation in this study was performed in JMP Pro from SAS. Below we provide instructions for replication with the necessary code. Data is provided separately as supplemental material.**

**EXPERIMENT 4**

**After importing Excel files into JMP, Bayesian model can be initiated.**

**Code for Peer choice estimation**

Choice(

Choice(

One Table( 1 ),

Subject ID( :subject ),

Choice Set ID( :Choice Set ),

Profile ID( :peer choice ),

Profile Effects(

:Shape,

:Fitness tracking,

:Name( "Health/heart monitoring" ),

:Brand,

:Price

),

Name( "Firth Bias-Adjusted Estimates" )(0),

Hierarchical Bayes( 1 ),

Number of Bayesian Iterations( 300000 ),

Respondents Are Allowed to Choose None( 1 ) Are Allowed to Choose None( 1 )

);

**Code for Own choice estimation**

Choice(

One Table( 1 ),

Subject ID( :subject ),

Choice Set ID( :Choice Set ),

Profile ID( :own choice),

Profile Effects(

:Shape,

:Fitness tracking,

:Name( "Health/heart monitoring" ),

:Brand,

:Price

),

Name( "Firth Bias-Adjusted Estimates" )(0),

Hierarchical Bayes( 1 ),

Number of Bayesian Iterations( 300000 ),

Effect Marginals( 1 ),

Confidence Limits( 1 )

);

**After the JMP computes estimates, please choose Save Bayes chain from the drop-down menu. The file with Bayes chain is used to compute utilities in validation sets using the code below. Variables needed for computation can be easily created in the datafile,they are specified below together with their formulas. Reported preference shares are means of variables with the same name.**

**Code for variable Set 1 utility A**

(:Name( "Mean of Shape[rectangular]" ) -

:Name( "Mean of Fitness tracking[No]" ) -

:Name( "Mean of Fitness tracking[Advanced]" )) +

:Name( "Mean of Health/heart monitoring[Basic]" ) +

:Name( "Mean of Brand[Apple]" ) + :Name( "Mean of Price[200]" )

**Code for variable Set 1 utility B**

((:Name( "Mean of Shape[rectangular]" ) -

:Name( "Mean of Fitness tracking[Advanced]" ) -

:Name( "Mean of Fitness tracking[No]" )) -

:Name( "Mean of Health/heart monitoring[Advanced]" ) -

:Name( "Mean of Health/heart monitoring[Basic]" )) +

:Name( "Mean of Brand[Apple]" ) + :Name( "Mean of Price[300]" )

**Code for variable Set 1 utility C**

:Name( "Mean of Shape[round classic]" ) +

:Name( "Mean of Fitness tracking[No]" ) +

:Name( "Mean of Health/heart monitoring[Basic]" ) + (

:Name( "Mean of Price[200]" ) - :Name( "Mean of Brand[Apple]" )

- :Name( "Mean of Brand[Garmin]" ))

**Code for variable Set 1 share of A**

Exp( :set 1 utility A ) / (Exp( :set 1 utility A )

+Exp( :set 1 utility B ) + Exp( :set 1 utility C ))

**Code for variable Set 1 share of B**

Exp( :set 1 utility B ) / (Exp( :set 1 utility A )

+Exp( :set 1 utility B ) + Exp( :set 1 utility C ))

**Code for variable Set 2 utility A**

((:Name( "Mean of Shape[rectangular]" ) -

:Name( "Mean of Fitness tracking[Advanced]" ) -

:Name( "Mean of Fitness tracking[No]" )) +

:Name( "Mean of Health/heart monitoring[Advanced]" ) +

:Name( "Mean of Brand[Apple]" )) -

:Name( "Mean of Price[200]" ) -

:Name( "Mean of Price[300]" )

**Code for variable Set 2 utility B**

(:Name( "Mean of Shape[round classic]" ) -

:Name( "Mean of Fitness tracking[Advanced]" ) -

:Name( "Mean of Fitness tracking[No]" ) -

:Name( "Mean of Health/heart monitoring[Advanced]" ) -

:Name( "Mean of Health/heart monitoring[Basic]" ) -

:Name( "Mean of Brand[Apple]" ) -

:Name( "Mean of Brand[Garmin]" )) +

:Name( "Mean of Price[300]" )

**Code for variable Set 2 utility C**

(((((-:Name( "Mean of Shape[rectangular]" )) -

:Name( "Mean of Shape[round classic]" )) +

:Name( "Mean of Fitness tracking[Advanced]" )) -

:Name( "Mean of Health/heart monitoring[Advanced]" ) -

:Name( "Mean of Health/heart monitoring[Basic]" )) +

:Name( "Mean of Brand[Garmin]" )) -

:Name( "Mean of Price[200]" ) -

:Name( "Mean of Price[300]" )

**Code for variable Set 2 share of A**

Exp( :set 2 utility A ) / (Exp( :set 2 utility A )

+Exp( :set 2 utility B ) + Exp( :set 2 utility C ))

**Code for variable Set 2 share of B**

Exp( :set 2 utility B ) / (Exp( :set 2 utility A )

+Exp( :set 2 utility B ) + Exp( :set 2 utility C ))

**Code for variable Set 3 utility A**

(:Name( "Mean of Shape[rectangular]" ) -

:Name( "Mean of Fitness tracking[Advanced]" ) -

:Name( "Mean of Fitness tracking[No]" ) -

:Name( "Mean of Health/heart monitoring[Advanced]" ) -

:Name( "Mean of Health/heart monitoring[Basic]" )) +

:Name( "Mean of Brand[Apple]" ) + :Name( "Mean of Price[300]" )

**Code for variable Set 3 utility B**

(((:Name( "Mean of Shape[rectangular]" ) -

:Name( "Mean of Fitness tracking[Advanced]" ) -

:Name( "Mean of Fitness tracking[No]" )) +

:Name( "Mean of Health/heart monitoring[Advanced]" )) +

:Name( "Mean of Brand[Apple]" )) - :Name( "Mean of Price[200]" ) -

:Name( "Mean of Price[300]" )

**Code for variable Set 3 utility C**

(:Name( "Mean of Shape[round classic]" ) -

:Name( "Mean of Fitness tracking[Advanced]" ) -

:Name( "Mean of Fitness tracking[No]" ) -

:Name( "Mean of Health/heart monitoring[Advanced]" ) -

:Name( "Mean of Health/heart monitoring[Basic]" ) -

:Name( "Mean of Brand[Apple]" ) - :Name( "Mean of Brand[Garmin]" ))

+ :Name( "Mean of Price[300]" )

**Code for variable Set 3 share of A**

Exp( :set 3 utility A ) / (Exp( :set 3 utility A )

+Exp( :set 3 utility B ) + Exp( :set 3 utility C ))

**Code for variable Set 3 share of B**

Exp( :set 3 utility B ) / (Exp( :set 3 utility A )

+Exp( :set 3 utility B ) + Exp( :set 3 utility C ))

**Code for variable Set 4 utiliity A**

(:Name( "Mean of Shape[round classic]" ) -

:Name( "Mean of Fitness tracking[Advanced]" ) -

:Name( "Mean of Fitness tracking[No]" ) -

:Name( "Mean of Health/heart monitoring[Advanced]" ) -

:Name( "Mean of Health/heart monitoring[Basic]" ) -

:Name( "Mean of Brand[Apple]" ) - :Name( "Mean of Brand[Garmin]" ))

+ :Name( "Mean of Price[300]" )

**Code for variable Set 4 utility B**

((:Name( "Mean of Shape[round classic]" ) +

:Name( "Mean of Fitness tracking[No]" ) +

:Name( "Mean of Health/heart monitoring[Basic]" )) -

:Name( "Mean of Brand[Apple]" ) - :Name( "Mean of Brand[Garmin]" ))

+ :Name( "Mean of Price[200]" )

**Code for variable Set 4 utility C**

(((((-:Name( "Mean of Shape[rectangular]" )) -

:Name( "Mean of Shape[round classic]" )) +

:Name( "Mean of Fitness tracking[Advanced]" )) -

:Name( "Mean of Health/heart monitoring[Advanced]" ) -

:Name( "Mean of Health/heart monitoring[Basic]" )) +

:Name( "Mean of Brand[Garmin]" )) - :Name( "Mean of Price[200]" )

-:Name( "Mean of Price[300]" )

**Code for variable Set 4 share of A**

Exp( :set 4 utility A ) / (Exp( :set 4 utility A )

+Exp( :set 4 utility B ) + Exp( :set 4 utility C ))

**Code for variable Set 4 share of B**

Exp( :set 4 utility B ) / (Exp( :set 4 utility A )

+Exp( :set 4 utility B ) + Exp( :set 4 utility C ))

**Code for variable Set 5 utility A**

(:Name( "Mean of Shape[rectangular]" ) -

:Name( "Mean of Fitness tracking[Advanced]" ) -

:Name( "Mean of Fitness tracking[No]" ) -

:Name( "Mean of Health/heart monitoring[Advanced]" ) -

:Name( "Mean of Health/heart monitoring[Basic]" )) +

:Name( "Mean of Brand[Apple]" ) + :Name( "Mean of Price[300]" )

**Code for variable Set 5 utility B**

(:Name( "Mean of Shape[round classic]" ) -

:Name( "Mean of Fitness tracking[Advanced]" ) -

:Name( "Mean of Fitness tracking[No]" ) -

:Name( "Mean of Health/heart monitoring[Advanced]" ) -

:Name( "Mean of Health/heart monitoring[Basic]" ) -

:Name( "Mean of Brand[Apple]" ) - :Name( "Mean of Brand[Garmin]" ))

+ :Name( "Mean of Price[300]" )

**Code for variable Set 5 utility C**

(:Name( "Mean of Shape[round classic]" ) +

:Name( "Mean of Fitness tracking[No]" ) +

:Name( "Mean of Health/heart monitoring[Basic]" ) +

:Name( "Mean of Price[200]" )) - :Name( "Mean of Brand[Apple]" ) -

:Name( "Mean of Brand[Garmin]" )

**Code for variable Set 5 share of A**

Exp( :set 5 utility A ) / (Exp( :set 5 utility A )

+Exp( :set 5 utility B ) + Exp( :set 5 utility C ))

**Code for variable Set 5 share of B**

Exp( :set 5 utility B ) / (Exp( :set 5 utility A )

+Exp( :set 5 utility B ) + Exp( :set 5 utility C ))

**Code for variable Set 6 utility A**

(:Name( "Mean of Shape[rectangular]" ) -

:Name( "Mean of Fitness tracking[Advanced]" ) -

:Name( "Mean of Fitness tracking[No]" ) -

:Name( "Mean of Health/heart monitoring[Advanced]" ) -

:Name( "Mean of Health/heart monitoring[Basic]" )) +

:Name( "Mean of Brand[Apple]" ) + :Name( "Mean of Price[300]" )

**Code for variable Set 6 utility B**

((:Name( "Mean of Shape[round classic]" ) +

:Name( "Mean of Fitness tracking[No]" ) +

:Name( "Mean of Health/heart monitoring[Basic]" )) -

:Name( "Mean of Brand[Apple]" ) - :Name( "Mean of Brand[Garmin]" ))

+ :Name( "Mean of Price[200]" )

**Code for variable Set 6 utility C**

(:Name( "Mean of Fitness tracking[Advanced]" ) -

:Name( "Mean of Shape[rectangular]" ) -

:Name( "Mean of Shape[round classic]" ) -

:Name( "Mean of Health/heart monitoring[Advanced]" ) -

:Name( "Mean of Health/heart monitoring[Basic]" ) -

:Name( "Mean of Price[200]" ) - :Name( "Mean of Price[300]" )) +

:Name( "Mean of Brand[Garmin]" )

**Code for variable Set 6 share of A**

Exp( :set 6 utility A ) / (Exp( :set 6 utility A )

+Exp( :set 6 utility B ) + Exp( :set 6 utility C ))

**Code for variable Set 6 share of B**

Exp( :set 6 utility B ) / (Exp( :set 6 utility A )

+Exp( :set 6 utility B ) + Exp( :set 6 utility C ))

**Code for variable Set 7 utility A**

(:Name( "Mean of Shape[rectangular]" ) -

:Name( "Mean of Fitness tracking[Advanced]" ) -

:Name( "Mean of Fitness tracking[No]" ) -

:Name( "Mean of Health/heart monitoring[Advanced]" ) -

:Name( "Mean of Health/heart monitoring[Basic]" )) +

:Name( "Mean of Brand[Apple]" ) + :Name( "Mean of Price[300]" )

**Code for variable Set 7 utility B**

((:Name( "Mean of Shape[rectangular]" ) -

:Name( "Mean of Fitness tracking[Advanced]" ) -

:Name( "Mean of Fitness tracking[No]" )) +

:Name( "Mean of Health/heart monitoring[Advanced]" ) +

:Name( "Mean of Brand[Apple]" )) - :Name( "Mean of Price[200]" ) -

:Name( "Mean of Price[300]" )

**Code for variable Set 7 utility C**

(:Name( "Mean of Fitness tracking[Advanced]" ) -

:Name( "Mean of Shape[rectangular]" ) -

:Name( "Mean of Shape[round classic]" ) -

:Name( "Mean of Health/heart monitoring[Advanced]" ) -

:Name( "Mean of Health/heart monitoring[Basic]" ) -

:Name( "Mean of Price[200]" ) - :Name( "Mean of Price[300]" )) +

:Name( "Mean of Brand[Garmin]" )

**Code for variable Set 7 share of A**

Exp( :set 7 utility A ) / (Exp( :set 7 utility A )

+Exp( :set 7 utility B ) + Exp( :set 7 utility C ))

**Code for variable Set 7 share of B**

Exp( :set 7 utility B ) / (Exp( :set 7 utility A )

+Exp( :set 7 utility B ) + Exp( :set 7 utility C ))

**Code for variable Set 8 utility A**

(:Name( "Mean of Shape[rectangular]" ) +

:Name( "Mean of Health/heart monitoring[Advanced]" ) +

:Name( "Mean of Brand[Apple]" )) -

:Name( "Mean of Fitness tracking[Advanced]" ) -

:Name( "Mean of Fitness tracking[No]" ) -

:Name( "Mean of Price[200]" ) - :Name( "Mean of Price[300]" )

**Code for variable Set 8 utility B**

(:Name( "Mean of Shape[round classic]" ) +

:Name( "Mean of Fitness tracking[No]" ) +

:Name( "Mean of Health/heart monitoring[Basic]" ) +

:Name( "Mean of Price[200]" )) - :Name( "Mean of Brand[Apple]" ) -

:Name( "Mean of Brand[Garmin]" )

**Code for variable Set 8 utility C**

:Name( "Mean of Fitness tracking[Advanced]" ) + (

:Name( "Mean of Brand[Garmin]" ) -

:Name( "Mean of Shape[rectangular]" ) -

:Name( "Mean of Shape[round classic]" ) -

:Name( "Mean of Health/heart monitoring[Advanced]" ) -

:Name( "Mean of Health/heart monitoring[Basic]" ) -

:Name( "Mean of Price[200]" ) - :Name( "Mean of Price[300]" ))

**Code for variable Set 8 share of A**

Exp( :set 8 utility A ) / (Exp( :set 8 utility A )

+Exp( :set 8 utility B ) + Exp( :set 8 utility C ))

**Code for variable Set 8 share of B**

Exp( :set 8 utility B ) / (Exp( :set 8 utility A )

+Exp( :set 8 utility B ) + Exp( :set 8 utility C))

**EXPERIMENT 1 AND EXPERIMENT 2**

**Code for Peer choice estimation**

Choice(

One Table( 1 ),

Subject ID( :subject ),

Choice Set ID( :Choice Set ),

Profile ID( :peer choice ),

Profile Effects( :price, :resolution, :remote, :use in hotels, :extra channels ),

Name( "Firth Bias-Adjusted Estimates" )(0),

Hierarchical Bayes( 1 ),

Number of Bayesian Iterations( 6000 ),

Respondents Are Allowed to Choose None( 1 )

);

**Code for Own choice**

Choice(

One Table( 1 ),

Subject ID( :subject ),

Choice Set ID( :Choice Set ),

Profile ID( :own choice ),

Profile Effects( :price, :resolution, :remote, :use in hotels, :extra channels ),

Name( "Firth Bias-Adjusted Estimates" )(0),

Hierarchical Bayes( 1 ),

Number of Bayesian Iterations( 300000 ),

);

**After the JMP computes estimates, please choose Save Bayes chain from the drop-down menu. The file with Bayes chain is used to compute utilities in validation sets using the code below. Variables needed for computation can be easily created in the datafile, they are specified below together with their formulas. Reported preference shares are means of variables with the same name.**

**Code for variable Set 6 utility of A**

((:Name( "Mean of price[40]" ) -

:Name( "Mean of resolution[1080]" )) + (

:Name( "Mean of remote[point anywhere]" ) -

:Name( "Mean of use in hotels[no]" ))) +

:Name( "Mean of extra channels[Amazon +DIRECTV]" )

**Code for variable Set 6 utility of B**

(:Name( "Mean of price[50]" ) + (

:Name( "Mean of resolution[1080]" ) -

:Name( "Mean of remote[none]" ) -

:Name( "Mean of remote[point anywhere]" ))) + (

:Name( "Mean of use in hotels[no]" ) -

:Name( "Mean of extra channels[all]" ) -

:Name( "Mean of extra channels[Amazon +DIRECTV]" ) -

:Name( "Mean of extra channels[AMAZON+FVG]" ))

**Code for variable Set 6 share of A**

Exp( :set 6 utility A ) / (Exp( :set 6 utility A )

+Exp( :set 6 utility B ))

**Code for variable Set 12 utility A**

:Name( "Mean of remote[point anywhere]" ) + (

:Name( "Mean of use in hotels[no]" ) -

:Name( "Mean of price[40]" ) - :Name( "Mean of price[50]" ) -

:Name( "Mean of price[70]" ) - :Name( "Mean of resolution[1080]" )

- :Name( "Mean of extra channels[all]" ) -

:Name( "Mean of extra channels[Amazon +DIRECTV]" ) -

:Name( "Mean of extra channels[AMAZON+FVG]" ))

**Code for variable Set 12 utility B**

:Name( "Mean of price[70]" ) + :Name( "Mean of resolution[1080]" ) +

:Name( "Mean of remote[none]" ) + (:Name( "Mean of extra channels[Amazon +DIRECTV]" )

- :Name( "Mean of use in hotels[no]" ))

**Code for variable Set 12 share of A**

Exp( :set 12 utility A ) / (Exp( :set 12 utility A )

+Exp( :set 12 utility B ))

**Code for variable Set 18 utility of** **A**

:Name( "Mean of resolution[1080]" ) -

:Name( "Mean of remote[none]" ) -

:Name( "Mean of remote[point anywhere]" ) -

:Name( "Mean of use in hotels[no]" ) -

:Name( "Mean of extra channels[all]" ) -

:Name( "Mean of extra channels[Amazon +DIRECTV]" ) -

:Name( "Mean of extra channels[AMAZON+FVG]" )

**Code for variable Set 18 utility of B**

:Name( "Mean of resolution[1080]" ) + :Name( "Mean of remote[none]" ) + (

:Name( "Mean of extra channels[AMAZON+FVG]" ) - :Name( "Mean of price[40]" )

- :Name( "Mean of price[50]" ) - :Name( "Mean of price[70]" ) -

:Name( "Mean of use in hotels[no]" ))

**Code for variable Set 18 share of A**

Exp( :set 18 utility A ) / (Exp( :set 18 utility A )

+Exp( :set 18 utility B ))

**Code for variable Real set utility A**

(:Name( "Mean of price[50]" ) + :Name( "Mean of resolution[1080]" ) + (

:Name( "Mean of remote[point anywhere]" ) -

:Name( "Mean of use in hotels[no]" ))) + :Name(

"Mean of extra channels[AMAZON+FVG]"

)

**Code for variable Real set utility B**

(:Name( "Mean of price[70]" ) - :Name( "Mean of resolution[1080]" )) +

:Name( "Mean of remote[none]" ) + :Name( "Mean of use in hotels[no]" ) +

:Name( "Mean of extra channels[AMAZON+FVG]" )

**Code for variable Real set utility C**

0 - :Name( "Mean of price[40]" ) - :Name( "Mean of price[50]" ) -

:Name( "Mean of price[70]" ) - :Name( "Mean of resolution[1080]" ) -

:Name( "Mean of remote[none]" ) - :Name( "Mean of remote[point anywhere]" )

-:Name( "Mean of use in hotels[no]" ) - :Name( "Mean of extra channels[all]" )

- :Name( "Mean of extra channels[Amazon +DIRECTV]" ) -

:Name( "Mean of extra channels[AMAZON+FVG]" )

**Code for variable Real set share A**

Exp( :real set utility A ) / ((Exp( :real set utility A )

+Exp( :real set utility B )) + Exp( :resl set utility C ))

**Code for variable Real set share B**

Exp( :real set utility B ) / ((Exp( :real set utility A )

+Exp( :real set utility B )) + Exp( :resl set utility C ))

**EXPERIMENT 3**

**Code for Peer choice estimation**

Choice(

One Table( 1 ),

Subject ID( :subject ),

Choice Set ID( :Choice Set ),

Profile ID( :peer choice ),

Profile Effects( :price, :resolution, :remote, :use in hotels, :extra channels ),

Name( "Firth Bias-Adjusted Estimates" )(0),

Hierarchical Bayes( 1 ),

Number of Bayesian Iterations( 6000 ),

Respondents Are Allowed to Choose None( 1 )

);

**Code for Own choice**

Choice(

One Table( 1 ),

Subject ID( :subject ),

Choice Set ID( :Choice Set ),

Profile ID( :own choice ),

Profile Effects( :price, :resolution, :remote, :use in hotels, :extra channels ),

Name( "Firth Bias-Adjusted Estimates" )(0),

Hierarchical Bayes( 1 ),

Number of Bayesian Iterations( 300000 ),

);

**After the JMP computes estimates, please choose Save Bayes chain from the drop-down menu. The file with Bayes chain is used to compute utilities in validation sets using the code below. Variables needed for computation can be easily created in the datafile, they are specified below together with their formulas. Reported preference shares are means of variables with the same name.**

**Code for variable Set 6 utility of A**

(((:Name( "Mean of heart rate[no]" ) + :Name( "Mean of GPS[no]" ) + (

:Name( "Mean of workout tracking[no]" ) - :Name(

"Mean of water resistance[splash]"

))) + :Name( "Mean of display and notifications[no display]" )) -

:Name( "Mean of battery life[14 days]" ) -

:Name( "Mean of battery life[5 days]" )) + :Name( "Mean of price[99.00 US$]" )

**Code for variable Set 6 utility of B**

(:Name( "Mean of heart rate[no]" ) + (:Name( "Mean of GPS[no]" ) -

:Name( "Mean of workout tracking[no]" ) - :Name(

"Mean of water resistance[splash]"

) - :Name( "Mean of display and notifications[no display]" ) -

:Name( "Mean of display and notifications[OLED +call text]" ))) +

:Name( "Mean of battery life[5 days]" ) + :Name( "Mean of price[79.00 US$]" )

**Code for variable Set 6 share of A**

Exp( :set 6 utility A ) / (Exp( :set 6 utility A )

+Exp( :set 6 utility B ))

**Code for variable Set 12 utility A**

(:Name( "Mean of heart rate[no]" ) + (:Name( "Mean of GPS[no]" ) -

:Name( "Mean of workout tracking[no]" ) - :Name(

"Mean of water resistance[splash]"

) - :Name( "Mean of display and notifications[no display]" ) -

:Name( "Mean of display and notifications[OLED +call text]" ))) +

:Name( "Mean of battery life[5 days]" ) + :Name( "Mean of price[79.00 US$]" )

**Code for variable Set 12 utility B**

(0 - :Name( "Mean of heart rate[no]" ) - :Name( "Mean of GPS[no]" )) +

:Name( "Mean of workout tracking[no]" ) + :Name(

"Mean of water resistance[splash]"

) + :Name( "Mean of display and notifications[no display]" ) +

:Name( "Mean of battery life[5 days]" ) + :Name( "Mean of price[79.00 US$]" )

**Code for variable Set 12 share of A**

Exp( :set 12 utility A ) / (Exp( :set 12 utility A )

+Exp( :set 12 utility B ))

**Code for variable Set 18 utility of** **A**

((0 - (:Name( "Mean of heart rate[no]" ) + :Name( "Mean of GPS[no]" ))) -

:Name( "Mean of workout tracking[no]" )) +

:Name( "Mean of water resistance[splash]" ) +

:Name( "Mean of display and notifications[OLED +call text]" ) +

:Name( "Mean of battery life[5 days]" ) + :Name( "Mean of price[99.00 US$]" )

**Code for variable Set 18 utility of B**

(-:Name( "Mean of heart rate[no]" )) + :Name( "Mean of GPS[no]" ) +

:Name( "Mean of workout tracking[no]" ) + :Name(

"Mean of water resistance[splash]"

) + :Name( "Mean of display and notifications[no display]" ) +

:Name( "Mean of battery life[5 days]" ) + :Name( "Mean of price[79.00 US$]" )

**Code for variable Set 18 share of A**

Exp( :set 18 utility A ) / (Exp( :set 18 utility A )

+Exp( :set 18 utility B ))

**Code for variable Real set utility A**

((:Name( "Mean of GPS[no]" ) + :Name( "Mean of heart rate[no]" ) +

:Name( "Mean of workout tracking[no]" ) + :Name(

"Mean of water resistance[splash]"

)) - :Name( "Mean of display and notifications[no display]" ) -

:Name( "Mean of display and notifications[OLED +call text]" )) +

:Name( "Mean of battery life[5 days]" ) + :Name( "Mean of price[99.00 US$]" )

**Code for variable Real set utility B**

(:Name( "Mean of heart rate[no]" ) + :Name( "Mean of GPS[no]" ) + (

:Name( "Mean of workout tracking[no]" ) - :Name(

"Mean of water resistance[splash]"

))) + :Name( "Mean of display and notifications[no display]" ) +

:Name( "Mean of battery life[5 days]" ) + :Name( "Mean of price[79.00 US$]" )

**Code for variable Real set utility C**

(((((-:Name( "Mean of heart rate[no]" )) - :Name( "Mean of GPS[no]" ) -

:Name( "Mean of workout tracking[no]" )) +

:Name( "Mean of water resistance[splash]" )) -

:Name( "Mean of display and notifications[no display]" ) -

:Name( "Mean of display and notifications[OLED +call text]" )) +

:Name( "Mean of battery life[5 days]" )) - :Name( "Mean of price[79.00 US$]" )

- :Name( "Mean of price[99.00 US$]" )

**Code for variable Real set share A**

Exp( :real set utility A ) / ((Exp( :real set utility A )

+Exp( :real set utility B )) + Exp( :resl set utility C ))

**Code for variable Real set share B**

Exp( :real set utility B ) / ((Exp( :real set utility A )

+Exp( :real set utility B )) + Exp( :resl set utility C ))
